# Trophic level and basal resource use of soil animals are hardly affected by local plant associations in abandoned arable land

**DOI:** 10.1002/ece3.6535

**Published:** 2020-07-07

**Authors:** Jörg‐Alfred Salamon, Janet Wissuwa, Thomas Frank, Stefan Scheu, Anton M. Potapov

**Affiliations:** ^1^ Institute of Ecology and Evolution & Field Station Schapen University of Veterinary Medicine Hannover Hannover Germany; ^2^ Department für Integrative Biologie und Biodiversitätsforschung Institut für Zoologie Universität für Bodenkultur Wien Wien Austria; ^3^ J.F. Blumenbach Institute of Zoology and Anthropology University of Goettingen Goettingen Germany; ^4^ Centre of Biodiversity and Sustainable Land Use Göttingen Germany; ^5^ A.N. Severtsov Institute of Ecology and Evolution Russian Academy of Sciences Moscow Russia

**Keywords:** aboveground‐belowground, *Bromus sterilis*, Formicidae, grassland, legume, macrofauna, *Medicaco sativa*, mesofauna, plant‐soil, stable isotopes, Staphylinidae, succession, *Taraxacum officinale*, trophic niche, δ^13^C, δ^15^N

## Abstract

Plants provide resources and shape the habitat of soil organisms thereby affecting the composition and functioning of soil communities. Effects of plants on soil communities are largely taxon‐dependent, but how different functional groups of herbaceous plants affect trophic niches of individual animal species in soil needs further investigation. Here, we studied the use of basal resources and trophic levels of dominating soil meso‐ and macrofauna using stable isotope ratios of carbon and nitrogen in arable fallow systems 3 and 14–16 years after abandonment. Animals were sampled from the rhizosphere of three plant species of different functional groups: a legume (*Medicaco sativa*), a nonlegume herb (*Taraxacum officinale*), and a grass (*Bromus sterilis*). We found virtually no consistent effects of plant identity on stable isotope composition of soil animals and on thirteen isotopic metrics that reflect general food‐web structure. However, in old fallows, the carbon isotope composition of some predatory macrofauna taxa had shifted closer to that of co‐occurring plants, which was particularly evident for *Lasius*, an aphid‐associated ant genus. Trophic levels and trophic‐chain lengths in food webs were similar across plant species and fallow ages. Overall, the results suggest that variations in local plant diversity of grassland communities may little affect the basal resources and the trophic level of prey consumed by individual species of meso‐ and macrofauna belowground. By contrast, successional changes in grassland communities are associated with shifts in the trophic niches of certain species, reflecting establishment of trophic interactions with time, which shapes the functioning and stability of soil food webs.

## Graphical abstract

Soil invertebrates depend on plants and plant residues as food source and habitat, but this link may be weaker than that for aboveground taxa and may have different mechanisms. We showed that in general, basal resources and trophic levels available for soil animal species are similar under different plants, but food‐web structure changes with the age of arable fallows.

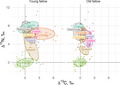

## 1 INTRODUCTION

The role of above‐belowground interactions for the functioning of terrestrial ecosystems is increasingly recognized (Grabmaier, Heigl, Eisenhauer, van der Heijden, & Zaller, 2014; Ohgushi, Wurst, & Johnson, 2018; Scheu, 2001; Wardle, Bardgett, & Klironomos, 2004). Plants, as organisms living both above and below the ground, are the main actors in these interactions. Via litter and roots they provide resources and habitat for soil organisms and thereby affect the composition, structure, and functioning of soil communities (Bonkowski, Villenave, & Griffiths, 2009; Wardle, 2002). In turn, soil biota control key ecosystem functions such as decomposition, nutrient mineralization, carbon sequestration, formation of soil structure, top‐down biological control, and, ultimately, soil fertility (Clemmensen et al., 2013; Filser, Faber, & Tiunov, 2016; Lavelle, Decaëns, & Aubert, 2006).

Plant diversity typically beneficially affects species directly linked to plants, such as above‐ground herbivores, while the effect on species indirectly linked to plants, such as detritivores in soil, is ambiguous (Korboulewsky, Perez, & Chauvat, 2016; Kuznetsova, Gomina, Smirnova, & Potapov, 2018; Scherber et al., 2010; Wardle, Yeates, Williamson, & Bonner, 2003). Although soil animal diversity has been shown to also increase with plant diversity, the effects are less pronounced than in aboveground species and vary among soil animal groups (Eisenhauer et al., 2010; Milcu, Partsch, Langel, & Scheu, 2006). Effects of aboveground vegetation on soil animals are often driven by the presence of certain plant species, rather than by plant diversity per se (Eisenhauer et al., 2010; Milcu et al., 2006; Salamon, Schaefer, Alphei, Schmid, & Scheu, 2004). For instance, in grassy arable fallows, soil meso‐ and macrofauna were shown to be more abundant under grass than under legume or herb species (Salamon, Wissuwa, Jagos, et al., 2011; Salamon, Wissuwa, Moder, Frank, 2011; Wissuwa, Salamon, & Frank, 2012). However, despite the composition of soil communities varying among plant species of different functional groups, they may perform similar functions and their feedbacks to plants may be little affected (Bezemer et al., 2010; Wardle et al., 2003). This emphasizes that multitrophic food‐web perspectives are needed to understand above‐belowground interaction effects on ecosystem functioning (Scherber et al., 2010).

Soil food webs rely on plants as the basal resource, either in the form of detritus or rhizodeposition by living roots (Goncharov & Tiunov, 2012; Moore et al., 2004). Most of soil meso‐ and macrofauna are not connected to plants via herbivory or feeding on litter, but rather via feeding on saprotrophic or root‐associated microorganisms (Potapov, Tiunov, & Scheu, 2019; Steffan & Dharampal, 2019; Swift, Heal, & Anderson, 1979). Soil animals are somewhat flexible in what they feed on, resulting in widespread trophic‐level and resource‐based omnivory in soil food webs (Digel, Curtsdotter, Riede, Klarner, & Brose, 2014). Some soil animal species may shift their trophic niches under ecosystem‐scale vegetation changes (Krause et al., 2019), but it is unclear if such shifts also occur within a single ecosystem due to local changes in plant associations.

Interactions in food webs develop and stabilize with time of species co‐existence, refining the trophic niches of species within communities (Giller, 1996; Korotkevich, Potapov, Tiunov, & Kuznetsova, 2018; Kuznetsova, 2006). To counteract biodiversity loss caused by intensive agricultural practices, arable land is increasingly left abandoned in Europe initiating secondary succession and the formation of grassy arable fallows (Hedlund et al., 2003). Such sites are progressively colonized by mixed grassy vegetation, with changes in soil biota not necessarily closely linked to changes in plant community composition and diversity (Frank & Reichhart, 2004; Hedlund et al., 2003; Holtkamp et al., 2008; Korthals, Smilauer, Van Dijk, & Van Der Putten, 2001; Scheu & Schulz, 1996). It is known that during the first 15–25 years of primary or secondary succession, the composition of soil fauna changes strongly and these changes are associated by extensive changes in the structure of soil food webs (Frouz et al., 2013; Holtkamp et al., 2008). In particular, soil food‐web development in grassy arable fallows results in increased biomass of lower trophic levels and the strengthening of the bacterial and fungal, but not the root energy channel (Holtkamp et al., 2008). However, it remains unknown how different plant species affect trophic niches of individual animal species during succession of grassy arable fallows and the associated soil food‐web development.

Studying trophic niches of soil animals is difficult since the soil matrix hampers direct observation. A number of methods including stable isotope, fatty acid, amino acid, and gut DNA analyses were suggested over last three decades to study trophic interactions among species (Nielsen, Clare, Hayden, Brett, & Kratina, 2017). Of these methods, stable isotope analysis provides a first‐line cost‐efficient assessment of trophic positions and basal resources of animal species in cryptic systems, such as in soil (Potapov et al., 2019; Tiunov, 2007). Bulk body tissue ^13^C/^12^C ratios reflect basal resources of the trophic chain animal species belong to (DeNiro & Epstein, 1978). In soil food webs, low ^13^C concentration indicates trophic link to freshly fixed plant carbon, for example, via herbivory, while high ^13^C concentration indicates trophic link to microbially processed carbon, for example, via feeding on microorganisms and detritus (Potapov et al., 2019). Bulk body tissue ^15^N/^14^N ratios reflect the trophic position of animal species in the trophic chain (DeNiro & Epstein, 1981). In soil food webs, high ^15^N concentration is related to high trophic level, but may also indicate a trophic link to soil organic matter or mycorrhizal fungi (Potapov et al., 2019). Stable isotope analysis allows insight into the structure of entire food webs by showing positions of species in relation to each other in “isotopic space” (Cucherousset & Villéger, 2015; Layman, Arrington, Montaña, & Post, 2007). For instance, high isotopic niche overlap in consumer species characterizes disturbed ecosystems, while in more developed, stable ecosystems isotopic niches of species are well differentiated (Korotkevich et al., 2018). However, stable isotope analysis has never been applied to assess trophic niches of soil animals under different plant species embedded in plant communities of different successional stages.

Here, we applied stable isotope analysis to study trophic niches of soil animals in the rhizosphere of three herbaceous plant species of contrasting functional groups: a legume (*Medicaco sativa*), a nonlegume herb (*Taraxacum officinale*), and a grass (*Bromus sterilis*). We studied both young (3 years after abandonment) and old arable fallows (14–16 years after abandonment) to inspect successional changes in trophic niches of soil animals under different plant species within mixed herbaceous vegetation. We hypothesized that trophic niches of soil animal species are different under different plant species, which is more evident in old fallows. We also hypothesized that differences in trophic niches among animal species will be smaller and intraspecific trophic niche variability will be higher in young than in old fallows, due to more refined trophic niches of species in more consolidated communities.

## 2 METHODS

### 2.1 Field collection

The study sites were located in the Marchfeld plain, an area of intensive agricultural production north‐east of Vienna characterized by a continental eastern European climate with mean annual temperature of 9.6°C and mean annual precipitation of 490 mm (Eitzinger, Zalud, & Alexandrov, 2001). In April 2009, soil samples were taken in three 3 and three 14–16 years old grassy arable fallows each including the following plant species: the legume *Medicaco sativa*, the nonlegume herb *Taraxacum officinale,* and the grass *Bromus sterilis*. Most of the fallows were established via spontaneous succession with some being additionally sown with a lucerne fallow seed mixture. All fallows were mown once a year. No detailed information about the former management practices on the fallows is available. Old fallows had higher plant diversity (+37%), fungal biomass (+150%), microbial biomass (+50%), water content (+25%), and C/N ratio (+50%) in soil but similar pH and basal respiration as young fallows (Salamon, Wissuwa, Jagos, et al., 2011; Salamon, Wissuwa, Moder, et al., 2011; Wissuwa et al., 2012). The dominating soil type in the sampling area was black earth (chernozem and parachernozem) (Table 1).

**TABLE 1:** Studied grassy arable fallows in the Marchfeld area, Austria

Site	Soil type	Coordinates	Size of the fallow (m^2^)	Fallow since
Young fallows (3 years) Early succession with lower plant diversity, fungal and microbial biomass, water content and C/N ratio in soil				
Site 1	Wet chernozem	48.225 N 16.871 E	1,709	2006
Site 2	Parachernozem	48.300 N 16.668 E	2,092	2006
Site 3	Parachernozem	48.307 N 16.490 E	1,969	2006
Old fallows (14–16 years) Late succession with higher plant diversity, fungal and microbial biomass, water content and C/N ratio in soil				
Site 4	Chernozem	48.341 N 16.723 E	1,168	1994
Site 5	Calcareous tilled soil	48.366 N 16.570 E	1,277	1996
Site 6	Chernozem	48.201 N 16.574 E	10,413	1993

Note

Characteristics of plant communities and soil properties were studied in Salamon, Wissuwa, Jagos, et al. (2011), Salamon, Wissuwa, Moder, et al. (2011) and Wissuwa et al. (2012).

In each of the six sites, three sampling plots (5 × 5 m) were randomly selected spaced by approximately 40 m to avoid spatial interdependence. Minimum distance of the plots to margins of the fallow was 15 m to minimize potential effects of neighboring ecosystems (i.e., arable fields or other grassy arable fallows). In April 2009, at each plot six plant individuals of each plant species (*M. sativa*, *T. officinale,* and *B. sterilis*) were harvested by cutting the plants 3 cm above the soil surface. All plants were harvested from the center of large patches (at least 1 m in diameter) of the target plant species to minimize edge effects and increase the likelihood that the plant species has been present on the spot for at least few years. The plant material of the six individual plants per species per plot was combined, cut into pieces of ca. 5 cm length and mixed for stable isotope analysis (see below), resulting in a total of 54 samples. Then, plant roots with the associated soil were sampled to a depth of 10 cm using a steel cylinder (5.6 × 5.6 cm). The soil material of the six individual plants per species per plot was combined to gain sufficient material for the extraction of soil meso‐ and macrofauna (in total 54 samples).

### 2.2 Animal and plant material

Soil meso‐ and macrofauna were extracted with a Berlese‐Tullgren funnel apparatus (Murphy, 1962) into saturated salt (NaCl) solution. The animals were stored in the solution at 4°C until identification and further processing. After animal extraction, roots of the target plant were picked by hand from the soil, washed, and cut into pieces. Identification of macro‐ and mesofauna was done under a microscope to genus or species level whenever possible. In total, 13 taxa were abundant in the study area and included in the analysis. Oribatida and Isopoda were identified to species level. Formicidae, Chilopoda, Staphylinidae (with the exception of Aleocharinae), and Collembola were identified to genus level. Due to a high number of juveniles, Julidae were identified to family level (Table 2).

**TABLE 2:** Studied soil animals classified according to their size class, trophic group, and taxonomic affiliation

Taxon	Δ^13^C, ‰		Δ^15^N, ‰	
	Young fallow	Old fallow	Young fallow	Old fallow
Mesofauna decomposers				
Collembola: *Protaphorura*	0.9 ± 0.7 (3)	0.6 ± 0.4 (7)	6.4 ± 0.6 (3)	6.1 ± 0.9 (7)
Collembola: *Lepidocyrtus*	1.1 ± 0.2 (2)	0.6 (1)	4.5 ± 1.8 (2)	4.4 (1)
Oribatida: *Philogalumna crassiclava*	2.8 ± 0.1 (2)	2.0 ± 0.5 (9)	8.0 ± 0.1 (2)	5.8 ± 1.6 (9)
Oribatida: *Punctoribates punctum*	2.3 ± 0.2 (7)	1.8 ± 0.2 (8)	5.9 ± 0.4 (7)	4.3 ± 0.4 (8)
Macrofauna decomposers				
Diplopoda: Julidae	1.4 ± 1.3 (12)	0.8 ± 0.9 (14)	3.2 ± 2.2 (12)	2.6 ± 0.7 (14)
Isopoda: *Oniscus asellus*	1.2 ± 0.9 (9)	1.4 ± 0.9 (4)	5.3 ± 0.9 (9)	5.4 ± 1.3 (4)
Macrofauna predators/omnivores				
Chilopoda: *Geophilus*	0.8 ± 1.7 (7)	−0.1 ± 0.3 (5)	7.8 ± 1.2 (7)	7.6 ± 0.6 (5)
Formicidae: *Lasius*	3.1 ± 2.6 (10)	0.3 ± 0.9 (28)	5.6 ± 0.4 (10)	5.5 ± 1.0 (28)
Formicidae: *Myrmica*	3.0 ± 1.9 (11)	1.5 ± 0.4 (6)	6.5 ± 0.8 (11)	6.2 ± 2.4 (6)
Formicidae: *Solenopsis*	3.8 ± 3.9 (4)	0.5 ± 0.5 (14)	7.9 ± 0.6 (4)	7.0 ± 0.5 (14)
Staphylinidae: Aleocharinae	0.6 ± 1.5 (11)	−0.2 ± 0.8 (16)	7.6 ± 1.7 (11)	6.6 ± 1.6 (16)
Staphylinidae: *Philonthus*	0.8 ± 1.0 (7)	0.7 ± 1.1 (5)	6.6 ± 1.6 (7)	8.4 ± 4.6 (5)
Staphylinidae: *Xantholinus*	0.8 ± 0.8 (6)	−1.8 (1)	9.1 ± 4.0 (6)	8.0 (1)

Note

Average baseline‐calibrated stable isotope composition (Δ^13^C and Δ^15^N values) was calculated for each taxon for young (3 years old) and old (14–16 years old) arable fallows across plant species. Means and standard deviations with the number of stable isotope measurements given in brackets.

Prior to carbon and nitrogen stable isotope analysis, root and shoot material was dried at 105°C for 24 hr and ground to powder (108 samples in total). Animals were transferred into tin capsules, dried at 60°C for 24 hr, and stored in a desiccator until analysis (209 samples in total). Appropriate amounts of dried and ground root and mixed leave/shoot material (800–1500 µg for ^13^C measurements and 3000–3400 µg for ^15^N measurements) and animal material (60–1300 µg) were weighed and wrapped into tin capsules. Samples were analyzed with a coupled system consisting of an elemental analyser (NA 1500, Carlo Erba, Milan, Italy) and a mass spectrometer (MAT 251, Finnigan, Bremen, Germany). The computer‐controlled system allowed on‐line measurement of stable isotope composition (^13^C, ^12^C, ^15^N, and ^14^N). Stable isotope composition was expressed using the δ notation with X (‰) = (R_sample_ − R_standard_)/R_standard_ × 1,000, where R_sample_ and R_standard_ represent the ^13^C/^12^C or ^15^N/^14^N ratios of samples and standard, respectively, and X displayed as δ^13^C or δ^15^N. As the primary standard for δ^13^C, PD belemnite (PDB) was used and for δ^15^N atmospheric nitrogen was used. Acetanilide (C_8_H_9_NO, Merck, Darmstadt) was used for internal calibration.

### 2.3 Statistical analysis

In most of the analyses, we were interested in trophic niches of soil animal species. To account for isotopic heterogeneity in basal resources, we subtracted site‐specific average δ^13^C and δ^15^N values across all plant samples from that of each animal sample, for example, for carbon baseline‐calibrated Δ^13^C = δ^13^C_animal_ − δ^13^C_plants, site‐specific average_ (Potapov et al., 2019).

All data computations and statistical analyses were performed in R 3.5.3 with Rstudio interface (Rstudio inc.). Changes in stable isotope composition and isotopic metrics were analyzed with linear mixed‐effects models using *lmer* in *lme4* package including sampling site as random effect (Bates, Mächler, Bolker, & Walker, 2015). In each analysis, we run a set of models by sequentially including factors and their interactions and then selecting the most parsimonious model based on the small sample size‐corrected Akaike's Information Criterion (AICc) using *AICc* in *AICcmodavg* package. The full R script is given in the Appendix S2 and model selection results also shown in Appendix S1 (Tables S1‐S5).

In the first analysis, we assessed variation in plant stable isotope composition. We tested the effect of plant species (*M. sativa*, *T. officinale*, and *B. sterilis*), plant compartment (roots, shoots), and fallow age (young, old fallows) on δ^13^C and δ^15^N values of plants.

In the second analysis, we assessed factors that may have affected baseline‐calibrated Δ^13^C and Δ^15^N values of soil animals, across all groups; the factors studied included stable isotope composition of plant compartment (roots and shoots), size group (macro‐ and mesofauna), trophic group (predators/omnivores and decomposers), fallow age (3, 14–16 years), and plant species (*M. sativa*, *T. officinale*, and *B. sterilis*). We included animal species identity as random effect in the model to account for statistical dependence of individuals coming from the same taxon. The model was selected in two steps: First, we tested if plant species, or Δ^13^C and Δ^15^N of plant roots, or Δ^13^C and Δ^15^N of plant shoots affected the Δ^13^C and Δ^15^N values in animal samples. Further factors (if any) were included in the next step of model selection with size group, trophic group, fallow age, and plant species as factors. Since we had no data on mesofauna predators, we tested the effects of trophic group and size group separately.

In the third analysis, we assessed the effect of plant species and fallow age on the entire food‐web structure. We approached this by calculating thirteen stable isotope metrics (Cucherousset & Villéger, 2015). The metrics reflect distribution of species in isotopic space. Single‐dimensional metrics included minimum, maximum, range, and mean (“IPos”) separately for Δ^13^C and Δ^15^N values in each community. Multidimensional metrics combined Δ^13^C and Δ^15^N values, and included isotopic richness (“IRic,” community convex hull area), isotopic divergence (“IDiv,” distribution of species within the community convex hull area), isotopic dispersion (“IDis,” deviation of species from the centroid), isotopic evenness (“IEve,” variation of isotopic distance among species), and isotopic uniqueness (“IUni,” closeness of species within the isotopic space, that is, inverse of isotopic redundancy). Baseline‐calibrated Δ^13^C and Δ^15^N values were scaled between 0 and 1 based on maximum and minimum across communities to ensure equal contribution of two isotope ratios in the multidimensional metrics calculation. The metrics were calculated for each site‐plant species combination using individual animal samples as the input data to the R script provided in Appendix S2 of Cucherousset and Villéger (2015). Site‐plant combinations were treated as communities and were used as replicates to inspect for differences in food‐web structure among plant species and fallow ages (see R script in Appendix S2). Model selection followed the procedures described above.

In the fourth analysis, we tested the effect of fallow age and plant species on baseline‐calibrated Δ^13^C and Δ^15^N values in individual species of soil animals. Here, only species that were present in five or six (out of the total of six) communities were included, with a minimum of two measurements in each community. Five species fitted the criteria. For these species, model selection followed the procedures described above.

In the fifth analysis, we assessed the effect of fallow age on the niche width in individual species of soil animals. First, we calculated standard deviations of baseline‐calibrated Δ^13^C and Δ^15^N values for each species on each site (only species with at least three measurements per site were included). Standard deviation values, as measure of niche width, were related to fallow age using linear mixed‐effects models including sampling site and species as random effects. Species identity was included as random effect due to insufficient number of replicates within each species.

In the text, we report least‐squares means from linear mixed‐effects models and corresponding confidence intervals. In figures and tables, we display regular means. Confidence intervals in figures were plotted using *ggpubr* extension for *ggplot2* package. All *p*‐values were calculated by Tukey contrasts using *glht* in *multcomp* package. We avoided using the *p*‐value threshold of .05 as evidence for scientific importance and avoided using the corresponding term “significant” following the recent call for “moving to a world beyond *p* < .05” (Wasserstein, Schirm, & Lazar, 2019). We report *p*‐values and effect sizes or means in the main text and in Appendix S2 R code to ensure transparency of our conclusions.

## 3 RESULTS

### 3.1 Stable isotope composition of plants

δ^13^C values of plants were related to plant species identity, plant compartment, and fallow age, as well as interactions between these factors. Mean δ^13^C values varied within 1‰ among plant species, being slightly higher in *M. sativa* (lsmean −27.5‰, 95% CI −28.1 to −26.9‰) than in *B. sterilis* (lsmean −27.8‰, 95% CI −28.4 to −27.2‰; *p* = .0034) and in *T. officinale* (lsmean −28.6‰, 95% CI −29.2 to −28.0‰; *p* = .0614). Shoots were 1–2‰ enriched in ^13^C compared to roots in *B. sterilis* and *M. sativa* in old fallows, but not in *M. sativa* in young fallows and in *T. officinale* in both young and old fallows (Figure S1). δ^15^N values of plants were not related to plant species identity or fallow age, but were 1‰ higher in shoots (lsmean 0.3‰, 95% CI −0.4 to 1.0‰) than in roots (lsmean −0.7‰, 95% CI −1.4 to 0.1‰; *p* < .0001; Figure S2).

### 3.2 Stable isotope composition of soil animal community

**FIGURE 1:**
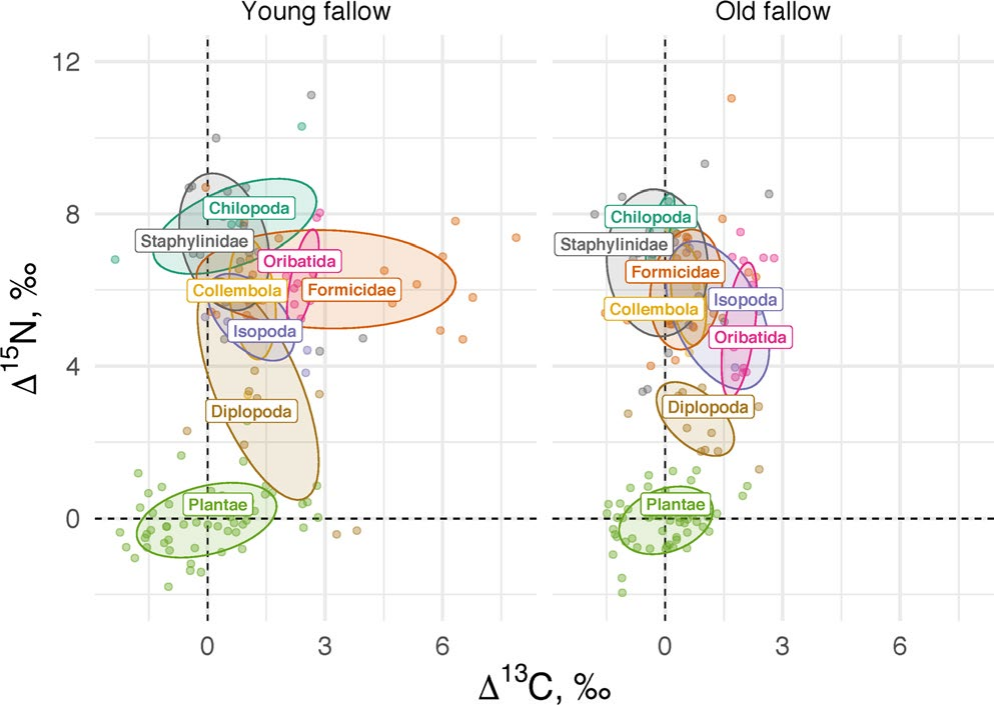
Baseline‐calibrated stable isotope composition (Δ^13^C and Δ^15^N values) of plants and soil animals in young (3 years old) and old arable fallows (14–16 years old). Data across sites are bulked. Each point represents one stable isotope measurement, two measurements of Staphylinidae with Δ^15^N > 15‰ are not shown. Ellipses are drawn at the 65% confidence level. Dashed lines represent plant isotopic baselines

Baseline‐calibrated Δ^13^C values of soil animals were not related to plant species identity or baseline‐calibrated Δ^13^C values of plant shoots or roots. The most parsimonious model explaining Δ^13^C values of soil animals included size group, fallow age, and their interaction (Table S1). Overall, Δ^13^C values of soil animals were higher in young than in old fallows (lsmean 1.7‰, 95% CI 0.5 to 6.9‰ and lsmean 0.9‰, 95% CI −0.3 to 2.1‰, respectively; *p* = .0388). Mesofauna had higher Δ^13^C values than macrofauna in old fallows (lsmean 1.5‰, 95% CI 0.1 to 2.8‰ and lsmean 0.3‰, 95% CI −0.9 to 1.5‰, respectively; *p* = .1340), while in young fallows Δ^13^C values of size groups were similar (lsmean 1.8‰, 95% CI 0.4 to 3.2‰ and lsmean 1.6‰, 95% CI 0.4 to 2.8‰, respectively; *p* = .9800) due to the presence of ^13^C‐enriched Formicidae (Figure 1).

Baseline‐calibrated Δ^15^N values of soil animals were not related to plant species identity or baseline‐calibrated Δ^15^N values of plant shoots or roots. The most parsimonious model explaining Δ^15^N values of soil animals included only trophic group (Table S2). A priori classified predators/omnivores (lsmean 7.1‰, 95% CI 6.1 to 8.1‰) had higher values than decomposers (lsmean 5.0‰, 95% CI 3.8 to 6.1‰; *p* = .0012). Fallow age was not selected for the model (Figure 1).

The most parsimonious models for all thirteen calculated stable isotope indices included only intercept and did not include plant species or fallow age reflecting no regular differences in species distribution on isotopic space with the studied factors (Table S3).

### 3.3 Stable isotope composition of individual species

**FIGURE 2:**
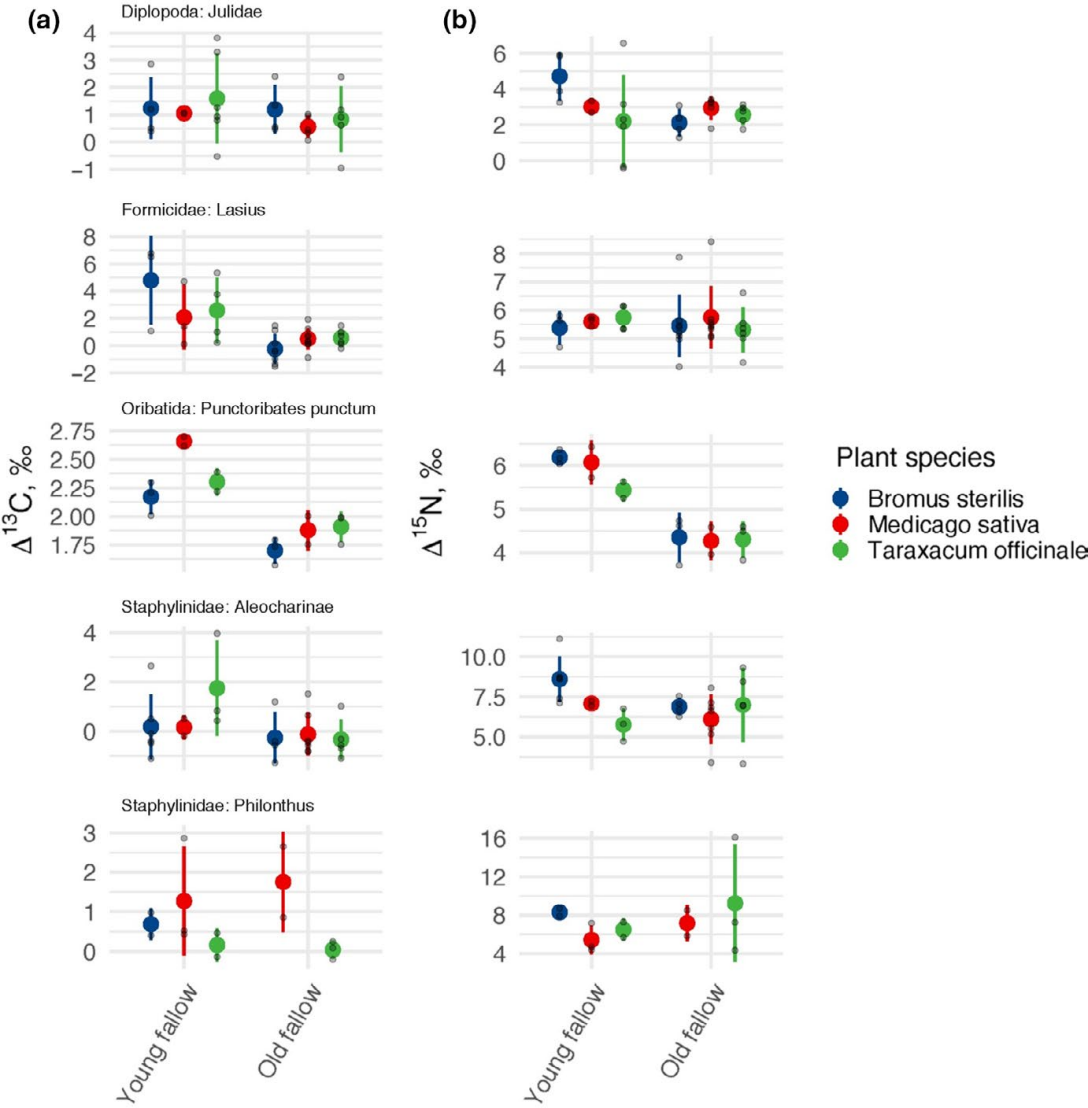
Baseline‐calibrated stable isotope composition (a: Δ^13^C values, b: Δ^15^N values) in individual soil animal taxa in soil of the three studied plant species in young and old fallows. Data across sites are bulked. Means and confidence intervals, individual measurements are shown with grey points

Five taxa were well‐represented across treatments allowing to run individual models: *Lasius* sp. (Formicidae), *P. punctum* (Oribatida), Aleocharinae, *Philonthus* sp. (Staphylinidae), and Julidae (Diplopoda). In two out of the five taxa, the most parsimonious model explaining baseline‐calibrated Δ^13^C values included fallow age, but not plant species (Table S4). In both cases, Δ^13^C values were higher in young than in old fallows (*Lasius* sp., lsmean 3.3‰, 95% CI 0.7 to 6.0‰ and lsmean 0.3‰, 95% CI −2.5 to 3.0‰, respectively; *p* = .0209; Aleocharinae, lsmean 0.7‰, 95% CI −0.5 to 1.9‰ and lsmean −0.1‰, 95% CI −1.5 to 1.2‰, respectively; *p* = .1378). In trend this was similar in *P. punctum*, but the model with fallow age had a higher AIC value than the zero model (Table S4). Δ^13^C values in the other taxa were not related to plant species or fallow age (Figure 2).

In four out of five well‐represented taxa, the most parsimonious model explaining the baseline‐calibrated Δ^15^N values did not include fallow age or plant species (Table S5). Δ^15^N values in Aleocharinae were related to plant species, being higher under *B. sterilis* (lsmean 7.9‰, 95% CI 6.8 to 9.1‰) than under *M. sativa* (lsmean 6.3‰, 95% CI −5.2 to 7.5‰; *p* = .0002) and *T. officinale* (lsmean 6.5‰, 95% CI 5.3 to 7.8‰; *p* = .1172), but this trend was evident mainly in young fallows (Figure 2). The model for *P. punctum* with fallow age had only slightly higher AIC value, than that of the zero model (Table S5); Δ^15^N values of this species were about 1.5‰ higher in young than in old fallows.

The ranges of baseline‐calibrated Δ^13^C values and Δ^15^N values were not related to fallow age across species, however, there were some young fallow sites where Aleocharinae, *Lasius* sp., and *Geophilus* sp. had very wide ranges of Δ^13^C values (Figure S6).

## 4 DISCUSSION

Using stable isotopes, we tested if herbaceous plant associations of different species affect the trophic level and basal resource use of soil animal species, and the overall distribution of trophic niches in soil animal communities, and if this varies with fallow age. Three plant species representing different plant functional groups, each abundant on arable fallows, were chosen: a legume (*M. sativa*), a nonlegume herb (*T. officinale*), and a grass (*B. sterilis*). Rejecting our first hypothesis, plant species identity did not have or had only little effect on Δ^13^C and Δ^15^N values of soil animals and the position of animal species in isotopic space relative to each other and plant resources. Only in part confirming our second hypothesis, fallow age affected the trophic niches of soil animals with some taxa having higher Δ^13^C values and higher Δ^13^C variation in young than in old fallows.

### 4.1 Soil food web under different plant species

We have chosen three functionally different plant species assuming that they will differentially affect the soil environment and therefore affect resources and trophic niches of soil animal species. The legume *M. sativa* forms mutualistic relationships with N‐fixing *Rhizobia*, which results in increased tissue nitrogen concentrations and thereby increased food quality of the litter (Zahran, 2001) as basal resource of the soil food web. The turf grass *B. sterilis* has high root density and thus provides pore structure and root‐derived resources for soil animals. The nonlegume herb *T. officinale* forms very different root system compared to the other two plant species with strongly developed taproot.

From previous studies, we learned that the variation in most soil properties, such as microbial biomass, water content, pH, and C/N ratio, is moderate under the studied plant species (Salamon, Wissuwa, Jagos, et al., 2011; Salamon, Wissuwa, Moder, et al., 2011; Wissuwa et al., 2012). However, community composition of soil meso‐ and macrofauna varied depending on the vegetation (Salamon, Wissuwa, Jagos, et al., 2011; Salamon, Wissuwa, Moder, et al., 2011; Wissuwa et al., 2012). We also found that overall densities of mites, saprophagous macrofauna, and root‐associated springtails were higher in *B. sterilis* soil than in soil under *M. sativa* and *T. officinale* (Salamon, Wissuwa, Jagos, et al., 2011; Salamon, Wissuwa, Moder, et al., 2011; Wissuwa et al., 2012), which was likely due to a higher fine root biomass and density of *B. sterilis* (Kutschera, 1960). As we showed in the present study, these alterations in soil animal communities were not associated with strong changes in Δ^13^C and Δ^15^N values (i.e., trophic positions and basal resources) of soil meso‐ and macrofauna, neither relative positions of species changed in isotopic space; rather, isotopic metrics of soil animals were similar across plant species. The only detected difference was higher Δ^15^N values in Aleocharinae in *B. sterilis* soil than in soil under *M. sativa* and *T. officinale*. This might reflect the ability of Aleocharine to switch from a more fungal to a more animal‐based diet in case of high prey availability—feeding on both is known for Aleocharinae (Lipkow & Betz, 2005).

Overall, the results indicate that differences in root architecture and tissue quality between plant species little affect the trophic niches of individual animal species, and their relative positions in the soil food web if these plants are embedded in plant communities of varying plant species composition. This may be related to the mobility of soil meso‐ and macrofauna, which are not restricted to a single plant association. However, it may also be related to similar trophic relationships among functional guilds forming under different herbaceous plants within an ecosystem.

### 4.2 Soil food‐web development in arable fallows

By comparing young and old fallows, we aimed at investigating shifts in trophic niches of soil animals with time in parallel to the succession of plant communities. Previous studies at our sites indicated that old fallows have higher plant diversity, fungal and microbial biomass, water content and C/N ratio than young fallows (Salamon, Wissuwa, Jagos, et al., 2011; Salamon, Wissuwa, Moder, et al., 2011; Wissuwa et al., 2012). Densities of most groups of soil animals were similar in young and old fallows, except for the decline in some mesopredators (Uropodina; Wissuwa et al., 2012) and increase in macropredators (Carabidae and Staphylinidae; Salamon, Wissuwa, Jagos, et al., 2011).

The soil animal community, especially predatory macrofauna, in old fallows had Δ^13^C values similar to those in plants, while in young fallows some of the animal taxa were enriched relative to plants (Figure 1). This shift could be explained either by (a) “legacy effect” due to differences in ^13^C concentration between crops cultivated prior to abandonment and current vegetation, or by (b) shift in trophic niches of soil animal species if the ^13^C concentration in crops and current vegetation was similar. The first few years after switching crop species, soil food webs extensively rely on old plant carbon stored in the soil and originating from preceding vegetation (Albers, Schaefer, & Scheu, 2006). Unfortunately, we cannot fully exclude this “legacy effect” as we do not have information on the crops cultivated on our study sites prior to abandonment. However, shifts in Δ^13^C values with fallow age were not uniform across animal groups, being high in Aleocharinae, ants (*Lasius* sp.) and centipedes (*Geophilus* sp.), but small in decomposer mesofauna (Collembola and Oribatida). Since decomposer mesofauna are more closely related to plants, such species‐specific shifts suggest that the change was, at least in part, based on shifts in trophic niches. If true, lower Δ^13^C values of macrofauna in old fallows, similar to those of plants, suggest increased importance of herbivory and decreased importance of microorganisms as basal resource in soil food webs in old compared to young fallows (Potapov et al., 2019). This is surprising, considering the about 150% increase in fungal biomass in old fallows (Salamon, Wissuwa, Jagos, et al., 2011). High Δ^13^C values of macrofauna in young arable fallows may reflect lower availability of new plant resources and trophic link to microbially decomposed old plant carbon with increased ^13^C concentration due to microbial decomposition, that is, “detrital shift” (Boström, Comstedt, & Ekblad, 2007; Potapov et al., 2019). Low Δ^13^C values of macrofauna in older (14–16 years) arable fallows may reflect stronger link to carbon of the current vegetation, for example, via ants and Staphylinidae feeding on aboveground or belowground herbivores. Unfortunately, we do not have data on the abundance of herbivores at our study sites to prove this assumption. Strong link of soil animals to the root‐associated mycorrhizal fungi associated with high fungal biomass may also explain low Δ^13^C values in soil animals in older fallows. However, since fungivores presumably feed little on arbuscular mycorrhizal fungi (Duhamel et al., 2013; Gange, 2000), more detailed studies on the mechanisms involved in plant—microorganism—microarthropod interactions in the rhizosphere are needed.

High variation in Δ^13^C values in young fallows in part was related to the high ^13^C enrichment of *Lasius* sp. In young fallows, this species had Δ^13^C values of about 3‰, while in old fallows Δ^13^C values were close to those in plants (i.e., zero), indicating a pronounced basal resource shift towards freshly fixed plant carbon (Potapov et al., 2019). *Lasius* spp. are known to be trophically linked to root‐associated aphids (Gilbert et al., 2014) and may develop more sustainable aphid colonies after abandonment of ploughing, that is, in old fallows, which may explain the corresponding low Δ^13^C values (i.e., feeding on herbivores).

In contrast to Δ^13^C, the overall range of Δ^15^N values in individual samples was similar in young and old fallows (15–17‰), and this was mainly due to the presence of highly ^15^N enriched staphylinid beetles in both. Also, the range of average taxon‐specific Δ^15^N values was similar in young and old fallows, but was much smaller than in individual samples (about 6‰). Assuming an enrichment in ^15^N of about 3.4‰ per trophic level (Post, 2002; Potapov et al., 2019), 6‰ is equivalent to about two trophic levels. Acting as herbivores or primary decomposers, Diplopoda were least enriched in ^15^N; Collembola, Oribatida, and Formicidae had intermediate Δ^15^N values, indicating a mixed plant—microbial—animal diet; Chilopoda and Staphylinidae were most enriched reflecting their role as predators (Appendix S1, Figure S4). In general, these positions fit well to previous descriptions of arable soil food webs (Albers et al., 2006; McNabb, Halaj, & Wise, 2001). Notably, we did not observe any consistent differences in trophic level of individual species or length of trophic chains between young and old fallows, suggesting temporal stability of trophic positions and food‐web structure on arable fallows in medium terms.

## 5 CONCLUSIONS

Little effect of plant identity on Δ^13^C and Δ^15^N values and position in isotopic space of soil meso‐ and macrofauna suggests that the trophic niches of soil animal species in arable fallows are insensitive to variations in local plant associations in mixed herbaceous plant communities. Results of this and our previous studies show that the effect of plant identity on soil communities is manifested via changing community composition, rather than changing trophic roles of species. This argues that variations in local plant diversity in grassland communities may only little affect the trophic niches of individual species belowground. On the other hand, the duration of ecosystem development affects trophic niches of individual taxa and is establishing interactions between species in soil food webs.

## CONFLICT OF INTEREST

None declared.

## AUTHOR CONTRIBUTIONS

**Jörg‐Alfred Salamon:** Conceptualization (equal); data curation (equal); formal analysis (equal); investigation (lead); methodology (equal); writing–original draft (equal); writing–review and editing (equal). **Janet Wissuwa:** Investigation (equal). **Thomas Frank:** Conceptualization (equal); funding acquisition (lead); methodology (equal); supervision (equal); writing–review and editing (equal). **Stefan Scheu:** Conceptualization (equal); supervision (equal); writing–review and editing (equal). **Anton M. Potapov:** Conceptualization (equal); data curation (equal); formal analysis (lead); visualization (lead); writing–original draft (lead); writing–review and editing (lead).

## Supporting information

Supplementary Material

Supplementary Material

Appendix S1

Appendix S2

## Data Availability

Raw data supporting the results are available from Dryad repository: https://doi.org/10.5061/dryad.5hqbzkh3h
