# Supplementary material for: Trophic level and basal resource use of soil animals are hardly affected by local plant associations in abandoned arable land
Source: Ecol Evol. 2020 Jul 7;10(15):8279–88. doi: 10.1002/ece3.6535 (PMC7417231; doi:10.1002/ece3.6535)
Supplement: Supplementary file 3 — Appendix S1 [file ECE3-10-8279-s003.docx]

**Supplementary materials**

**Trophic level and basal resource use of soil animals are hardly affected by local plant associations in abandoned arable land**

Jörg-Alfred Salamon^1,2^, Janet Wissuwa^2^, Thomas Frank^2^, Stefan Scheu^3,4^ and Anton M. Potapov^3,5*^

^1^University of Veterinary Medicine Hannover, Institute of Animal Ecology & Field Station Schapen, Bünteweg 17 d, 30559 Hannover, Germany

^2^Institut für Zoologie, Department für Integrative Biologie und Biodiversitätsforschung, Universität für Bodenkultur Wien, Gregor-Mendel-Straße 33, 1180 Wien, Austria

^3^J.F. Blumenbach Institute of Zoology and Anthropology, University of Goettingen, Untere Karspüle 2, 37073 Goettingen, Germany

^4^Centre of Biodiversity and Sustainable Land Use, Von-Siebold-Str. 8, 37075 Göttingen, Germany

^5^A.N. Severtsov Institute of Ecology and Evolution, Russian Academy of Sciences, Leninsky Prospect 33, 119071 Moscow, Russia

***Anton M. Potapov

e-mail: [potapov.msu@gmail.com](mailto:potapov.msu@gmail.com)

**Table of contents**

- Supplementary Tables S1-S5
- Supplementary Figures S1-S6
- Data analysis: annotated R script (provided as a separate file)
- Data files: “Data_raw_tabular_plants.csv”, “metrics_Salamon.csv” (provided as separate files)

**Table S1.** Final model selection for Δ^13^C values across all animals. Model with the lowest small sample size-corrected Akaike’s Information Criterion (AICc) was selected.

| Model formula | AICc |
| --- | --- |
| **D13C ~ 1 + (Fallow_age + Size_group)^2 + (1 \| Site) + (1 \| Taxon)** | **692.9** |
| D13C ~ 1 + Fallow_age + Size_group + (1 \| Site) + (1 \| Taxon) | 694.4 |
| D13C ~ 1 + Fallow_age + (1 \| Site) + (1 \| Taxon) | 694.8 |
| D13C ~ 1 + Fallow_age + Size_group + Plant species + (1 \| Site) + (1 \| Taxon) | 701.1 |
| D13C ~ 1 + Fallow_age + Plant species + (1 \| Site) + (1 \| Taxon) | 701.5 |
| D13C ~ 1 + (Fallow_age + Plant species)^2 + (1 \| Site) + (1 \| Taxon) | 702.8 |
| D13C ~ 1 + (Fallow_age + Size_group + Plant species)^2 + (1 \| Site) + (1 \| Taxon) | 704.4 |

**Table S2.** Final model selection for Δ^15^N values across all animals. Model with the lowest small sample size-corrected Akaike’s Information Criterion (AICc) was selected.

| Model formula | AICc |
| --- | --- |
| **D15N ~ 1 + Trophic_group + (1 \| Site) + (1 \| Taxon)** | **781.8** |
| D15N ~ 1 + Trophic_group + Fallow_age + (1 \| Site) + (1 \| Taxon) | 782.4 |
| D15N ~ 1 + (Trophic_group + Fallow_age)^2 + (1 \| Site) + (1 \| Taxon) | 783.9 |
| D15N ~ 1 + Trophic_group + Plant species + (1 \| Site) + (1 \| Taxon) | 785.4 |
| D15N ~ 1 + Trophic_group + Plant species + Fallow_age + (1 \| Site) + (1 \| Taxon) | 786.2 |
| D15N ~ 1 + (Trophic_group + Plant species)^2 + (1 \| Site) + (1 \| Taxon) | 787.6 |
| D15N ~ 1 + (Trophic_group + Plant species + Fallow_age)^2 + (1 \| Site) + (1 \| Taxon) | 790.4 |

**Table S3.** Model selection for thirteen isotopic metrics. For each metric, model with the lowest small sample size-corrected Akaike’s Information Criterion (AICc) was selected.

| Metric, model formula | AICc |
| --- | --- |
| min_d15N |  |
| **value ~ 1 + (1 \| Site)** | **-30.0929743** |
| value ~ 1 + Fallow_age + (1 \| Site) | -22.9084731 |
| value ~ 1 + Plant species + (1 \| Site) | -14.2099369 |
| value ~ 1 + Plant species + Fallow_age + (1 \| Site) | -5.75170945 |
| min_d13C |  |
| **value ~ 1 + (1 \| Site)** | **-26.5830969** |
| value ~ 1 + Fallow_age + (1 \| Site) | -20.076545 |
| value ~ 1 + Plant species + (1 \| Site) | -14.7600923 |
| value ~ 1 + Plant species + Fallow_age + (1 \| Site) | -6.97981417 |
| max_d15N |  |
| **value ~ 1 + (1 \| Site)** | **-5.74144559** |
| value ~ 1 + Fallow_age + (1 \| Site) | 0.528099138 |
| value ~ 1 + Plant species + (1 \| Site) | 6.043525485 |
| value ~ 1 + Plant species + Fallow_age + (1 \| Site) | 13.58679649 |
| max_d13C |  |
| **value ~ 1 + (1 \| Site)** | **-6.06855914** |
| value ~ 1 + Fallow_age + (1 \| Site) | -2.53406627 |
| value ~ 1 + Plant species + (1 \| Site) | 6.426201763 |
| value ~ 1 + Plant species + Fallow_age + (1 \| Site) | 11.23442091 |
| range_d15N |  |
| **value ~ 1 + (1 \| Site)** | **1.557634678** |
| value ~ 1 + Fallow_age + (1 \| Site) | 7.645145638 |
| value ~ 1 + Plant species + (1 \| Site) | 12.11336446 |
| value ~ 1 + Plant species + Fallow_age + (1 \| Site) | 19.47460176 |
| range_d13C |  |
| **value ~ 1 + (1 \| Site)** | **3.825251202** |
| value ~ 1 + Fallow_age + (1 \| Site) | 8.143847548 |
| value ~ 1 + Plant species + (1 \| Site) | 12.72633082 |
| value ~ 1 + Plant species + Fallow_age + (1 \| Site) | 18.31865344 |
| IPos_d15N |  |
| **value ~ 1 + (1 \| Site)** | **-56.3077018** |
| value ~ 1 + Fallow_age + (1 \| Site) | -47.95493 |
| value ~ 1 + Plant species + (1 \| Site) | -37.7806264 |
| value ~ 1 + Plant species + Fallow_age + (1 \| Site) | -28.1541283 |
| IPos_d13C |  |
| **value ~ 1 + (1 \| Site)** | **-36.8309924** |
| value ~ 1 + Fallow_age + (1 \| Site) | -32.5463274 |
| value ~ 1 + Plant species + (1 \| Site) | -19.0457844 |
| value ~ 1 + Plant species + Fallow_age + (1 \| Site) | -13.4873931 |
| IRic |  |
| **value ~ 1 + (1 \| Site)** | **-57.175964** |
| value ~ 1 + Fallow_age + (1 \| Site) | -49.7038448 |
| value ~ 1 + Plant species + (1 \| Site) | -41.3942322 |
| value ~ 1 + Plant species + Fallow_age + (1 \| Site) | -32.6483867 |
| IDiv |  |
| **value ~ 1 + (1 \| Site)** | **-38.3077151** |
| value ~ 1 + Fallow_age + (1 \| Site) | -29.5938105 |
| value ~ 1 + Plant species + (1 \| Site) | -24.225504 |
| value ~ 1 + Plant species + Fallow_age + (1 \| Site) | -14.14578 |
| IDis |  |
| **value ~ 1 + (1 \| Site)** | **-12.3114552** |
| value ~ 1 + Fallow_age + (1 \| Site) | -5.55932137 |
| value ~ 1 + Plant species + (1 \| Site) | 0.71640395 |
| value ~ 1 + Plant species + Fallow_age + (1 \| Site) | 8.742264076 |
| IEve |  |
| **value ~ 1 + (1 \| Site)** | **-33.1155118** |
| value ~ 1 + Fallow_age + (1 \| Site) | -24.8472505 |
| value ~ 1 + Plant species + (1 \| Site) | -17.9527914 |
| value ~ 1 + Plant species + Fallow_age + (1 \| Site) | -8.4108038 |
| IUni |  |
| **value ~ 1 + (1 \| Site)** | **-18.7609977** |
| value ~ 1 + Fallow_age + (1 \| Site) | -11.7383285 |
| value ~ 1 + Plant species + (1 \| Site) | -10.1469917 |
| value ~ 1 + Plant species + Fallow_age + (1 \| Site) | -1.85059623 |

**Table S4.** Model selection for Δ^13^C values in individual animal taxa. For each taxon, model with the lowest small sample size-corrected Akaike’s Information Criterion (AICc) was selected.

| Taxon, model formula | AICc |
| --- | --- |
| Julidae |  |
| **D13C ~ 1 + (1 \| Site)** | **85.4548** |
| D13C ~ 1 + Fallow_age + (1 \| Site) | 86.51758 |
| D13C ~ 1 + Plant species + (1 \| Site) | 89.31535 |
| D13C ~ 1 + Plant species + Fallow_age + (1 \| Site) | 91.41368 |
| Lasius |  |
| **D13C ~ 1 + Fallow_age + (1 \| Site)** | **130.0802** |
| D13C ~ 1 + Plant species + Fallow_age + (1 \| Site) | 133.872 |
| D13C ~ 1 + (1 \| Site) | 134.0437 |
| D13C ~ 1 + Plant species + (1 \| Site) | 137.1547 |
| Punctoribates punctum |  |
| **D13C ~ 1 + (1 \| Site)** | **8.79583** |
| D13C ~ 1 + Fallow_age + (1 \| Site) | 11.06737 |
| D13C ~ 1 + Plant species + (1 \| Site) | 13.72706 |
| D13C ~ 1 + Plant species + Fallow_age + (1 \| Site) | 17.9963 |
| Aleocharinae |  |
| **D13C ~ 1 + Fallow_age + (1 \| Site)** | **91.86065** |
| D13C ~ 1 + (1 \| Site) | 91.86773 |
| D13C ~ 1 + Plant species + (1 \| Site) | 95.31335 |
| D13C ~ 1 + Plant species + Fallow_age + (1 \| Site) | 95.53201 |
| Philonthus |  |
| **D13C ~ 1 + (1 \| Site)** | **42.62397** |
| D13C ~ 1 + Plant species + (1 \| Site) | 45.06055 |
| D13C ~ 1 + Fallow_age + (1 \| Site) | 46.52093 |
| D13C ~ 1 + Plant species + Fallow_age + (1 \| Site) | 52.44649 |

**Table S5.** Model selection for Δ^15^N values in individual animal taxa. For each taxon, model with the lowest small sample size-corrected Akaike’s Information Criterion (AICc) was selected.

| Taxon, model formula | AICc |
| --- | --- |
| Julidae |  |
| **D15N ~ 1 + (1 \| Site)** | **97.86614** |
| D15N ~ 1 + Fallow_age + (1 \| Site) | 98.05704 |
| D15N ~ 1 + Plant species + (1 \| Site) | 101.0041 |
| D15N ~ 1 + Plant species + Fallow_age + (1 \| Site) | 101.8473 |
| Lasius |  |
| **D15N ~ 1 + (1 \| Site)** | **88.14947** |
| D15N ~ 1 + Fallow_age + (1 \| Site) | 91.13117 |
| D15N ~ 1 + Plant species + (1 \| Site) | 93.51722 |
| D15N ~ 1 + Plant species + Fallow_age + (1 \| Site) | 96.85107 |
| Punctoribates punctum |  |
| **D15N ~ 1 + (1 \| Site)** | **30.31934** |
| D15N ~ 1 + Fallow_age + (1 \| Site) | 30.32378 |
| D15N ~ 1 + Plant species + (1 \| Site) | 38.66757 |
| D15N ~ 1 + Plant species + Fallow_age + (1 \| Site) | 40.72179 |
| Aleocharinae |  |
| **D15N ~ 1 + Plant species + (1 \| Site)** | **109.4224** |
| D15N ~ 1 + Fallow_age + (1 \| Site) | 110.6927 |
| D15N ~ 1 + Plant species + Fallow_age + (1 \| Site) | 111.0126 |
| D15N ~ 1 + (1 \| Site) | 111.1157 |
| Philonthus |  |
| **D15N ~ 1 + (1 \| Site)** | **67.66878** |
| D15N ~ 1 + Fallow_age + (1 \| Site) | 68.37962 |
| D15N ~ 1 + Plant species + (1 \| Site) | 70.50689 |
| D15N ~ 1 + Plant species + Fallow_age + (1 \| Site) | 74.96185 |


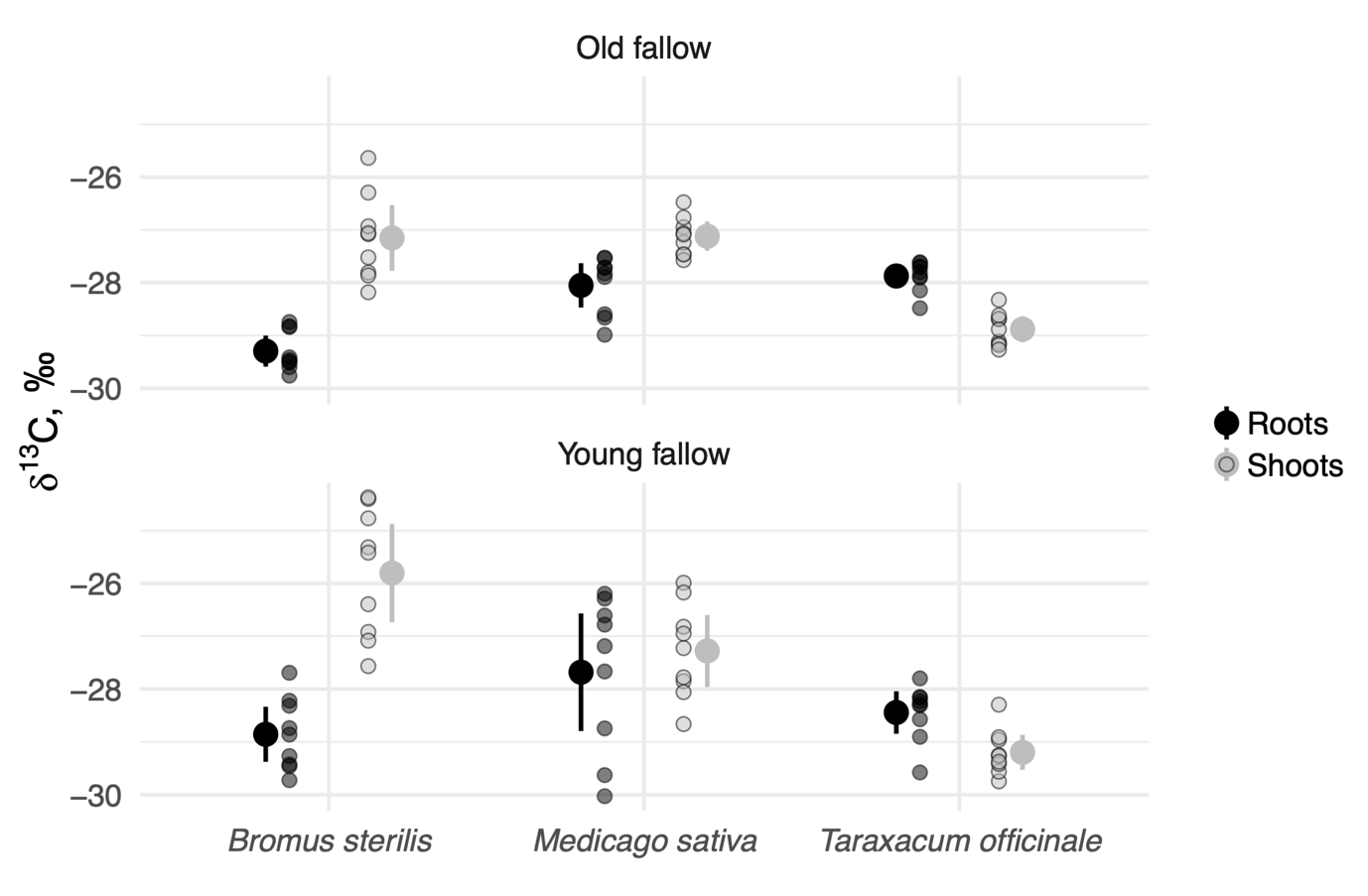


**Figure S1.** Carbon stable isotope composition (δ^13^C values) of plant roots and shoots in three studied species in young and old fallows. Data across sites are bulked together. Means and confidence intervals, individual measurements are shown with small points.


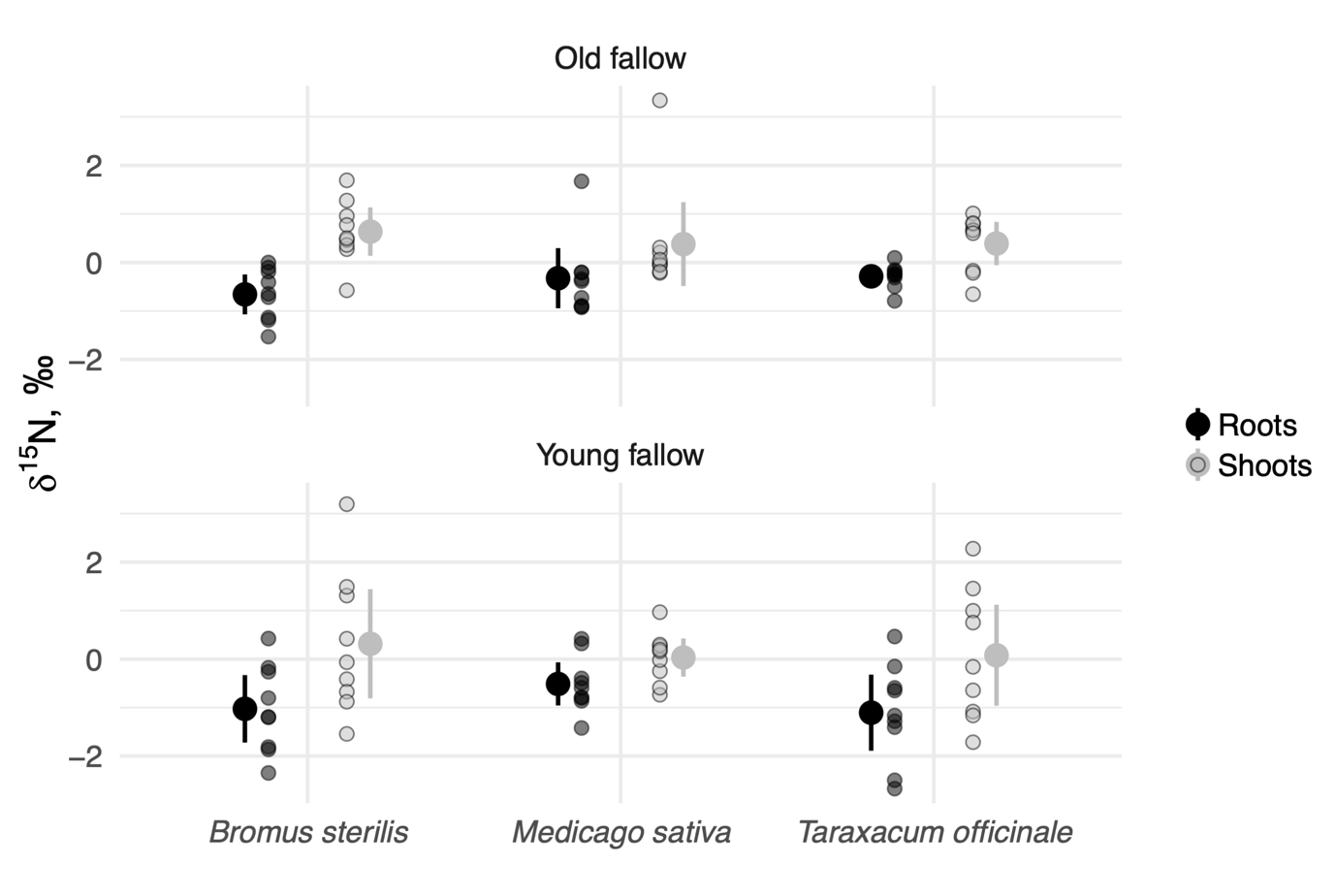


**Figure S2.** Nitrogen stable isotope composition (δ^15^N values) of plant roots and shoots in three studied species in young and old fallows. Data across sites are bulked together. Means and confidence intervals, individual measurements are shown with small points.


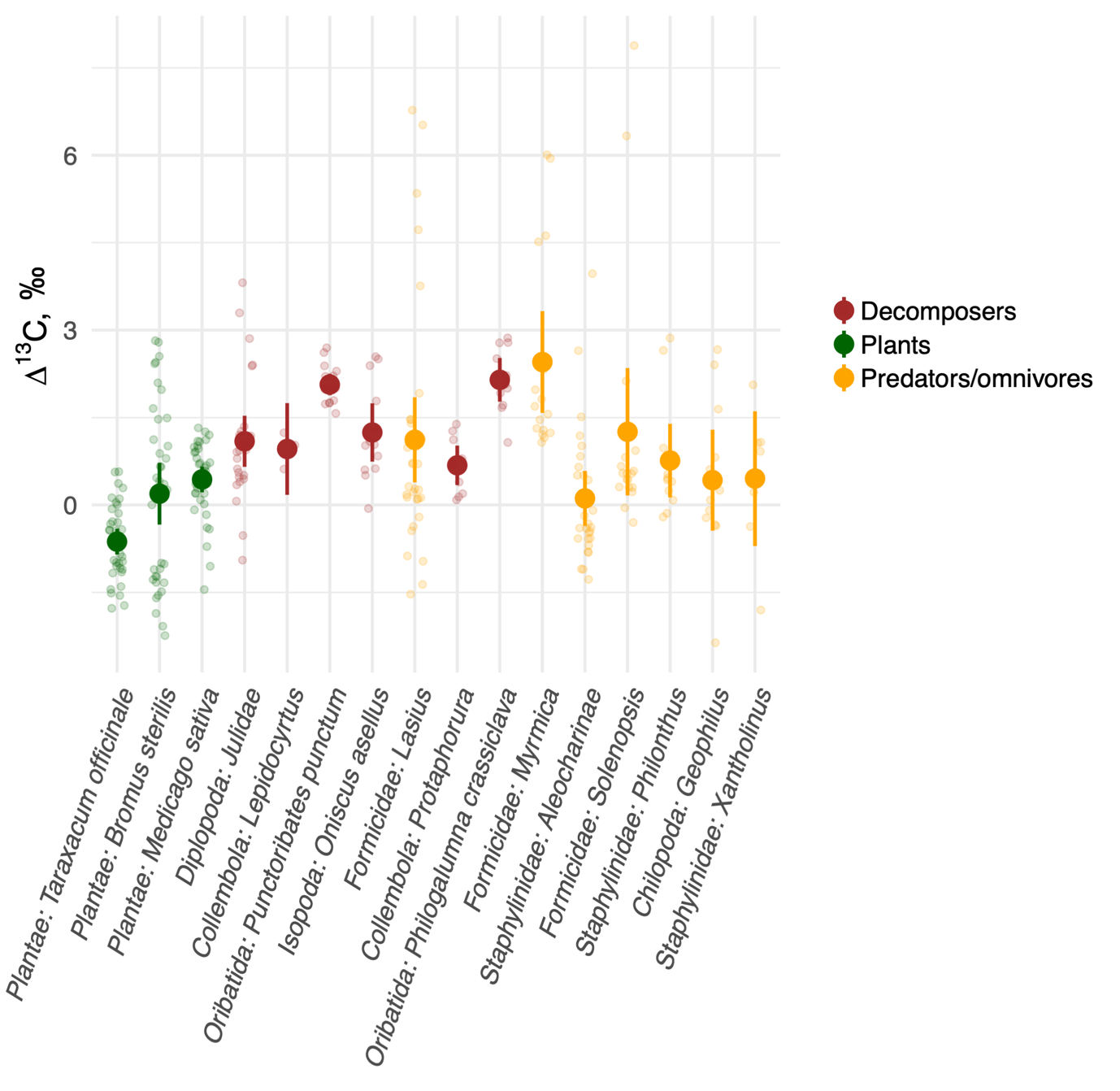


**Figure S3.** Carbon stable isotope composition (δ^13^C values) of soil animal species. Data across sites are bulked together. Means and confidence intervals, individual measurements are shown with small points. Taxa are ordered according to their mean δ^15^N values (see Fig. S4).


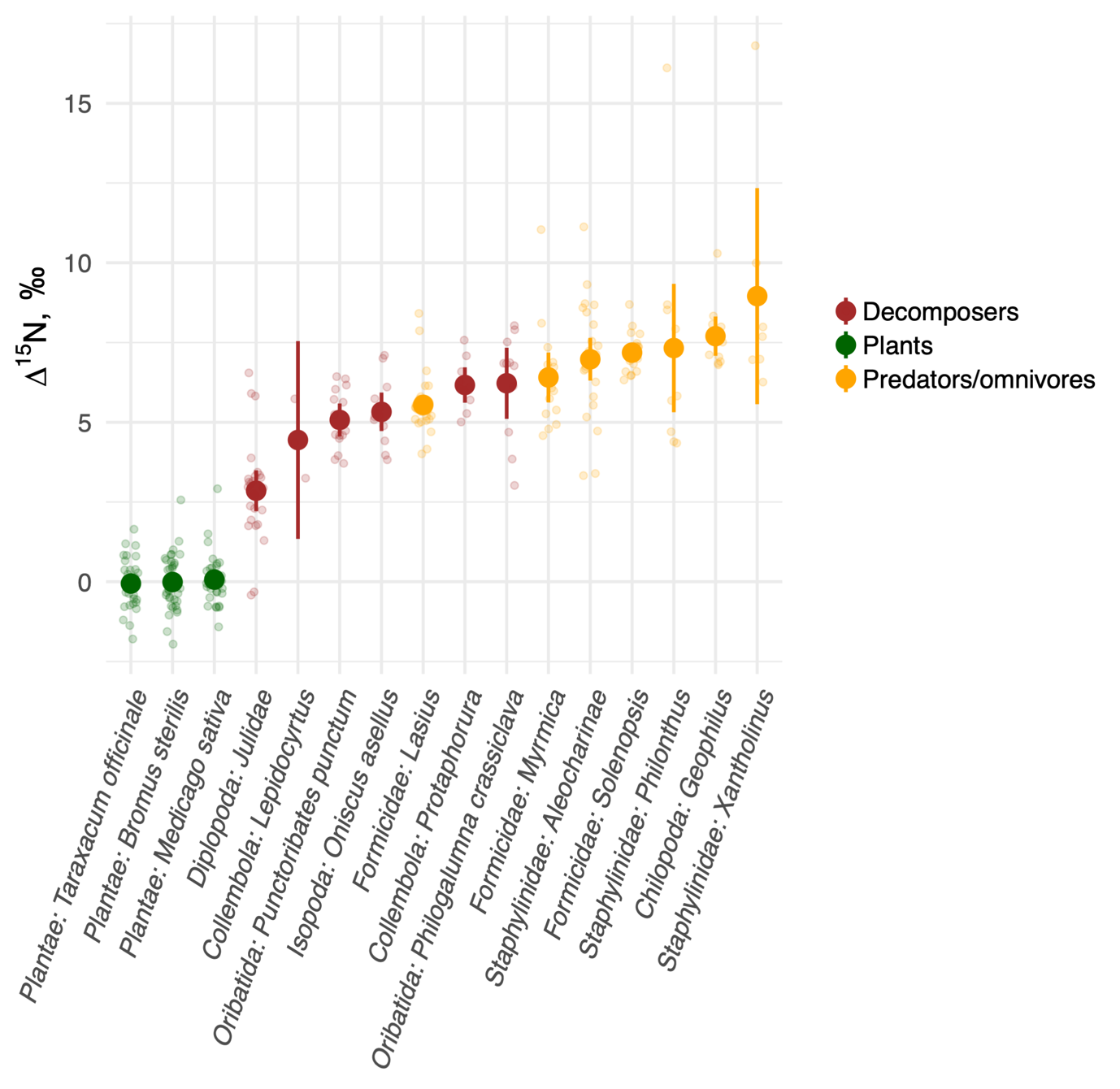


**Figure S4.** Nitrogen stable isotope composition (δ^15^N values) of soil animal species. Data across sites are bulked together. Means and confidence intervals, individual measurements are shown with small points.


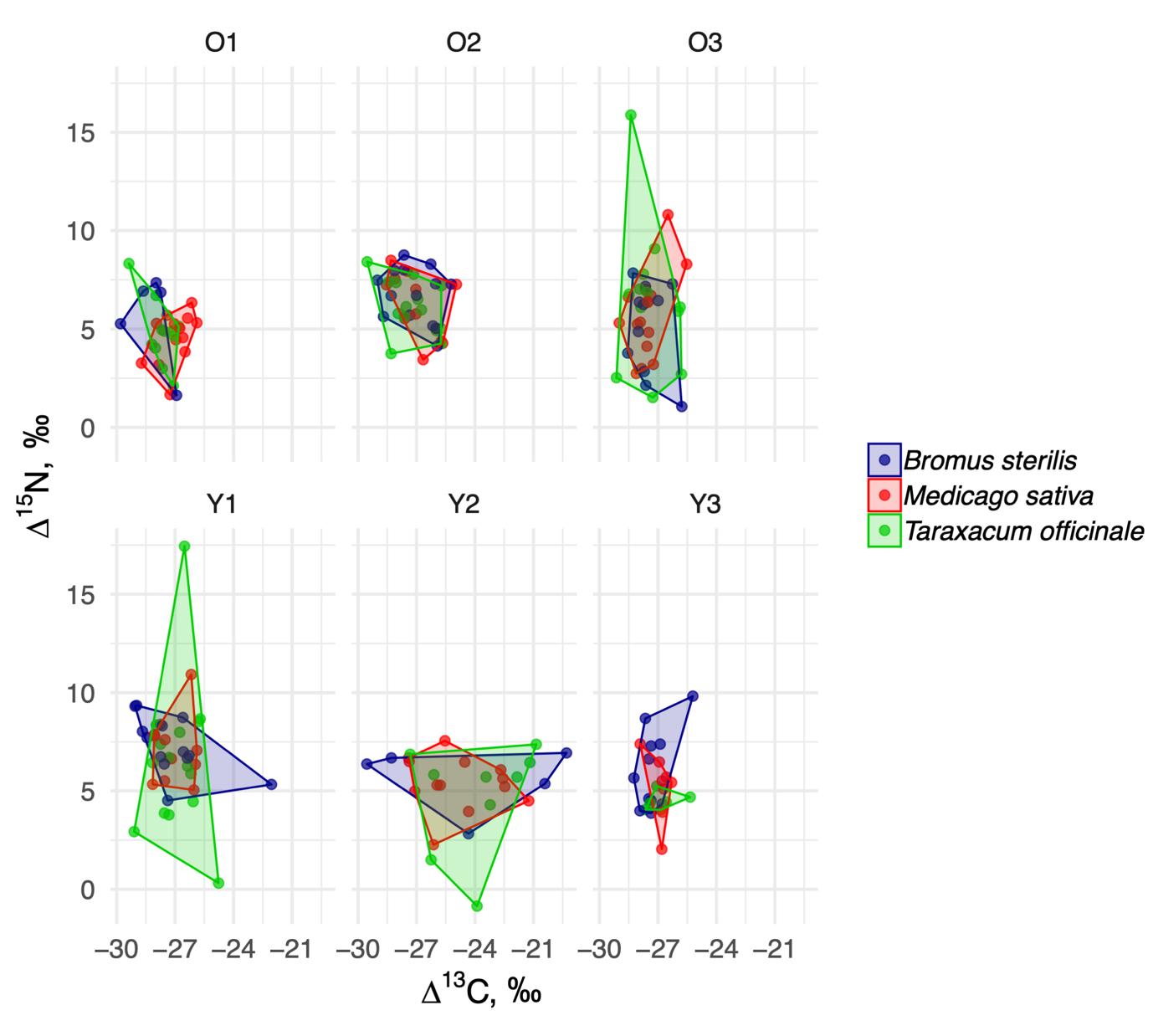


**Figure S5.** Stable isotope composition (δ^13^C and δ^15^N values) of soil animals on different sites and under different plant species. Young arable fallows (Y1, Y2, Y3) are 3 years old, old arable fallows (O1, O2, O3) are 14-16 years old. Each point is a stable isotope measurement. Convex hulls connect measurements with most extreme isotopic values.

**Figure S6.** Standard deviations of baseline-calibrated stable isotope composition (A: Δ^13^C values, B: Δ^15^N values) in individual species of soil animals on young and old fallows. Means and confidence intervals, standard deviations in each species on each site are shown with small points.
