# Supplementary material for: Trophic level and basal resource use of soil animals are hardly affected by local plant associations in abandoned arable land
Source: Ecol Evol. 2020 Jul 7;10(15):8279–88. doi: 10.1002/ece3.6535 (PMC7417231; doi:10.1002/ece3.6535)
Supplement: Supplementary file 4 — Appendix S2 [file ECE3-10-8279-s004.docx]

Data analysis Salamon et al. Trophic level and basal resource use of soil animals are hardly affected by local plant associations in abandoned arable land

Anton Potapov

21/07/2019

### 1: Install and load libraries -----------------------------------------------------------------

# List the packages
used.packages <- c("tidyverse", # a collection of packages, including ggplot, dplyr and more
 "reshape2", # data transformation
 "reshape", # data transformation
 "lme4",
 "lmerTest",
 "lsmeans",
 "LMERConvenienceFunctions",
 "AICcmodavg",
 "vegan",
 "multcomp"
 )

# Install and/or update all packages if needed
# lapply(used.packages, install.packages, character.only = TRUE)
# lapply(used.packages, update.packages, character.only = TRUE)

# Load all packages in the working space
lapply(used.packages, library, character.only = TRUE)

## ── Attaching packages …

# Load cleaned data
Data_raw_tabular_plants <- read.csv(file = "Data_raw_tabular_plants.csv", header = T,
 dec = ".", # the decimal point
 sep = ",") # the separator symbol

# Calculate baseline-calibrated values for roots and shoots
Data_raw_tabular_plants$D13Croots <- Data_raw_tabular_plants$d13Croots - Data_raw_tabular_plants$d13Cbaseline
Data_raw_tabular_plants$D13Cshoots <- Data_raw_tabular_plants$d13Cshoots - Data_raw_tabular_plants$d13Cbaseline
Data_raw_tabular_plants$D15Nroots <- Data_raw_tabular_plants$d15Nroots - Data_raw_tabular_plants$d15Nbaseline
Data_raw_tabular_plants$D15Nshoots <- Data_raw_tabular_plants$d15Nshoots - Data_raw_tabular_plants$d15Nbaseline


### 2: Plant isotopic composition variation ----------------------------------------------------------------------------------

### Load data for plants into 'fast-name' object
A <- Data_raw_tabular_plants %>%
 dplyr::select(c(15,16,18,17,13,1:5,7:14,19,20)) %>%
 filter(Group=="Plantae")

### LMM model for d13C of plants depending root/shoot, species and fallow age
model13C_plant1 <- lmer(d13C~(1|Site),A)
model13C_plant2 <- lmer(d13C~-1+bodypart+(1|Site),A)
model13C_plant3 <- lmer(d13C~-1+Fallow_age+(1|Site),A)
model13C_plant4 <- lmer(d13C~-1+Taxon+(1|Site),A)
model13C_plant5 <- lmer(d13C~-1+Taxon+bodypart+(1|Site),A)
model13C_plant6 <- lmer(d13C~-1+Taxon+Fallow_age+(1|Site),A)
model13C_plant7 <- lmer(d13C~-1+bodypart+Fallow_age+(1|Site),A)
model13C_plant8 <- lmer(d13C~-1+Taxon+bodypart+Fallow_age+(1|Site),A)
model13C_plant9 <- lmer(d13C~-1+(Taxon+bodypart)^2+(1|Site),A)
model13C_plant10 <- lmer(d13C~-1+(Taxon+Fallow_age)^2+(1|Site),A)
model13C_plant11 <- lmer(d13C~-1+(bodypart+Fallow_age)^2+(1|Site),A)
model13C_plant12 <- lmer(d13C~-1+(Taxon+bodypart+Fallow_age)^2+(1|Site),A)

model13C_iso_list <- c(model13C_plant1,model13C_plant2,model13C_plant3,model13C_plant4,model13C_plant5,
 model13C_plant6,model13C_plant7,model13C_plant8,model13C_plant9,model13C_plant10,model13C_plant11,model13C_plant12)

# Compare models with AICc
# The model with all factors and interactions is selected (model13C_plant12)
AICc <- lapply(model13C_iso_list,AICc)
Model <- lapply(model13C_iso_list,function(x) as.character(c(formula(x))))
model13Cselect_plant <- cbind(Model,AICc)
model13Cselect_plant

## Model
## [1,] "d13C ~ (1 | Site)"
## [2,] "d13C ~ -1 + bodypart + (1 | Site)"
## [3,] "d13C ~ -1 + Fallow_age + (1 | Site)"
## [4,] "d13C ~ -1 + Taxon + (1 | Site)"
## [5,] "d13C ~ -1 + Taxon + bodypart + (1 | Site)"
## [6,] "d13C ~ -1 + Taxon + Fallow_age + (1 | Site)"
## [7,] "d13C ~ -1 + bodypart + Fallow_age + (1 | Site)"
## [8,] "d13C ~ -1 + Taxon + bodypart + Fallow_age + (1 | Site)"
## [9,] "d13C ~ -1 + (Taxon + bodypart)^2 + (1 | Site)"
## [10,] "d13C ~ -1 + (Taxon + Fallow_age)^2 + (1 | Site)"
## [11,] "d13C ~ -1 + (bodypart + Fallow_age)^2 + (1 | Site)"
## [12,] "d13C ~ -1 + (Taxon + bodypart + Fallow_age)^2 + (1 | Site)"
## AICc
## [1,] 352.8112
## [2,] 342.8182
## [3,] 354.6911
## [4,] 341.1818
## [5,] 328.9514
## [6,] 343.1477
## [7,] 344.7405
## [8,] 330.9621
## [9,] 254.8206
## [10,] 339.9265
## [11,] 346.6171
## [12,] 246.3434

# Write the model estimates with confidence intervals
selected_model <- model13C_iso_list[which.min(AICc)][[1]] # the model selected
rownames(anova(selected_model))

## [1] "Taxon" "bodypart" "Fallow_age"
## [4] "Taxon:bodypart" "Taxon:Fallow_age" "bodypart:Fallow_age"

lsmeans(selected_model,rownames(anova(selected_model))[1], adjust="none")

## NOTE: Results may be misleading due to involvement in interactions

## Taxon lsmean SE df lower.CL upper.CL
## Bromus sterilis -27.8 0.233 5.27 -28.4 -27.2
## Medicago sativa -27.5 0.233 5.27 -28.1 -26.9
## Taraxacum officinale -28.6 0.233 5.27 -29.2 -28.0
##
## Results are averaged over the levels of: bodypart, Fallow_age
## Degrees-of-freedom method: kenward-roger
## Confidence level used: 0.95

lsmeans(selected_model,rownames(anova(selected_model))[2], adjust="none")

## NOTE: Results may be misleading due to involvement in interactions

## bodypart lsmean SE df lower.CL upper.CL
## Roots -28.4 0.225 4.62 -29.0 -27.8
## Shoots -27.6 0.225 4.62 -28.2 -27.0
##
## Results are averaged over the levels of: Taxon, Fallow_age
## Degrees-of-freedom method: kenward-roger
## Confidence level used: 0.95

lsmeans(selected_model,rownames(anova(selected_model))[3], adjust="none")

## NOTE: Results may be misleading due to involvement in interactions

## Fallow_age lsmean SE df lower.CL upper.CL
## Young fallow -27.9 0.307 4 -28.7 -27.0
## Old fallow -28.1 0.307 4 -28.9 -27.2
##
## Results are averaged over the levels of: Taxon, bodypart
## Degrees-of-freedom method: kenward-roger
## Confidence level used: 0.95

lsmeans <- as.data.frame(lsmeans(selected_model,rownames(anova(selected_model))[c(1:3)], adjust="none"))
lsmeans[,-c(length(lsmeans)-5:length(lsmeans))] <- round(lsmeans[,-c(length(lsmeans)-5:length(lsmeans))],2)
lsmeans

## Taxon bodypart Fallow_age lsmean SE df lower.CL
## 1 Bromus sterilis Roots Young fallow -28.68 0.35 6.7 -29.52
## 2 Medicago sativa Roots Young fallow -27.87 0.35 6.7 -28.70
## 3 Taraxacum officinale Roots Young fallow -28.43 0.35 6.7 -29.27
## 4 Bromus sterilis Shoots Young fallow -25.98 0.35 6.7 -26.81
## 5 Medicago sativa Shoots Young fallow -27.09 0.35 6.7 -27.93
## 6 Taraxacum officinale Shoots Young fallow -29.21 0.35 6.7 -30.04
## 7 Bromus sterilis Roots Old fallow -29.47 0.35 6.7 -30.30
## 8 Medicago sativa Roots Old fallow -27.87 0.35 6.7 -28.70
## 9 Taraxacum officinale Roots Old fallow -27.88 0.35 6.7 -28.72
## 10 Bromus sterilis Shoots Old fallow -26.98 0.35 6.7 -27.81
## 11 Medicago sativa Shoots Old fallow -27.31 0.35 6.7 -28.14
## 12 Taraxacum officinale Shoots Old fallow -28.87 0.35 6.7 -29.71
## upper.CL
## 1 -27.85
## 2 -27.03
## 3 -27.60
## 4 -25.14
## 5 -26.26
## 6 -28.37
## 7 -28.64
## 8 -27.03
## 9 -27.05
## 10 -26.14
## 11 -26.47
## 12 -28.04

write.csv(lsmeans,"model13Cselect_summaryPlant.csv")

# Test the differences with Tukey contrasts
posthoc <- glht(selected_model, linfct=mcp(Taxon="Tukey"))

## Warning in mcp2matrix(model, linfct = linfct): covariate interactions found
## -- default contrast might be inappropriate

summary(posthoc)

##
## Simultaneous Tests for General Linear Hypotheses
##
## Multiple Comparisons of Means: Tukey Contrasts
##
##
## Fit: lmer(formula = d13C ~ -1 + (Taxon + bodypart + Fallow_age)^2 +
## (1 | Site), data = A)
##
## Linear Hypotheses:
## Estimate Std. Error z value
## Medicago sativa - Bromus sterilis == 0 0.8156 0.2511 3.249
## Taraxacum officinale - Bromus sterilis == 0 0.2478 0.2511 0.987
## Taraxacum officinale - Medicago sativa == 0 -0.5678 0.2511 -2.262
## Pr(>|z|)
## Medicago sativa - Bromus sterilis == 0 0.00321 **
## Taraxacum officinale - Bromus sterilis == 0 0.58495
## Taraxacum officinale - Medicago sativa == 0 0.06131 .
## ---
## Signif. codes: 0 '***' 0.001 '**' 0.01 '*' 0.05 '.' 0.1 ' ' 1
## (Adjusted p values reported -- single-step method)

### LMM model for D15N of plants depending root/shoot, species and fallow age
model15N_plant1 <- lmer(d15N~-1+(1|Site),A)
model15N_plant2 <- lmer(d15N~-1+bodypart+(1|Site),A)
model15N_plant3 <- lmer(d15N~-1+Fallow_age+(1|Site),A)
model15N_plant4 <- lmer(d15N~-1+Taxon+(1|Site),A)
model15N_plant5 <- lmer(d15N~-1+Taxon+bodypart+(1|Site),A)
model15N_plant6 <- lmer(d15N~-1+Taxon+Fallow_age+(1|Site),A)
model15N_plant7 <- lmer(d15N~-1+bodypart+Fallow_age+(1|Site),A)
model15N_plant8 <- lmer(d15N~-1+Taxon+bodypart+Fallow_age+(1|Site),A)
model15N_plant9 <- lmer(d15N~-1+(Taxon+bodypart)^2+(1|Site),A)
model15N_plant10 <- lmer(d15N~-1+(Taxon+Fallow_age)^2+(1|Site),A)
model15N_plant11 <- lmer(d15N~-1+(bodypart+Fallow_age)^2+(1|Site),A)
model15N_plant12 <- lmer(d15N~-1+(Taxon+bodypart+Fallow_age)^2+(1|Site),A)

model15N_iso_list <- c(model15N_plant1,model15N_plant2,model15N_plant3,model15N_plant4,model15N_plant5,
 model15N_plant6,model15N_plant7,model15N_plant8,model15N_plant9,model15N_plant10,model15N_plant11,model15N_plant12)

# Compare models with AICc
# The model with bodypart only is selected (model15N_plant2)
AICc <- lapply(model15N_iso_list,AICc)
Model <- lapply(model15N_iso_list,function(x) as.character(c(formula(x))))
model15Nselect_plant <- cbind(Model,AICc)
model15Nselect_plant

## Model
## [1,] "d15N ~ -1 + (1 | Site)"
## [2,] "d15N ~ -1 + bodypart + (1 | Site)"
## [3,] "d15N ~ -1 + Fallow_age + (1 | Site)"
## [4,] "d15N ~ -1 + Taxon + (1 | Site)"
## [5,] "d15N ~ -1 + Taxon + bodypart + (1 | Site)"
## [6,] "d15N ~ -1 + Taxon + Fallow_age + (1 | Site)"
## [7,] "d15N ~ -1 + bodypart + Fallow_age + (1 | Site)"
## [8,] "d15N ~ -1 + Taxon + bodypart + Fallow_age + (1 | Site)"
## [9,] "d15N ~ -1 + (Taxon + bodypart)^2 + (1 | Site)"
## [10,] "d15N ~ -1 + (Taxon + Fallow_age)^2 + (1 | Site)"
## [11,] "d15N ~ -1 + (bodypart + Fallow_age)^2 + (1 | Site)"
## [12,] "d15N ~ -1 + (Taxon + bodypart + Fallow_age)^2 + (1 | Site)"
## AICc
## [1,] 278.1339
## [2,] 237.0991
## [3,] 281.5747
## [4,] 287.7886
## [5,] 245.0284
## [6,] 288.8352
## [7,] 238.1022
## [8,] 246.1198
## [9,] 245.7139
## [10,] 293.252
## [11,] 241.0159
## [12,] 255.3928

# Write the model estimates with confidence intervals
selected_model <- model15N_iso_list[which.min(AICc)][[1]] # the model selected
rownames(anova(selected_model))

## [1] "bodypart"

lsmeans(selected_model,rownames(anova(selected_model))[1], adjust="none")

## bodypart lsmean SE df lower.CL upper.CL
## Roots -0.651 0.287 5.49 -1.368 0.0662
## Shoots 0.306 0.287 5.49 -0.411 1.0233
##
## Degrees-of-freedom method: kenward-roger
## Confidence level used: 0.95

lsmeans <- as.data.frame(lsmeans(selected_model,rownames(anova(selected_model))[1], adjust="none"))
lsmeans[,-c(length(lsmeans)-5:length(lsmeans))] <- round(lsmeans[,-c(length(lsmeans)-5:length(lsmeans))],2)
lsmeans

## bodypart lsmean SE df lower.CL upper.CL
## 1 Roots -0.65 0.29 5.49 -1.37 0.07
## 2 Shoots 0.31 0.29 5.49 -0.41 1.02

write.csv(lsmeans,"model15Nselect_summaryPlant.csv")

# Test the differences with Tukey contrasts
posthoc <- glht(selected_model, linfct=mcp(bodypart="Tukey"))
summary(posthoc)

##
## Simultaneous Tests for General Linear Hypotheses
##
## Multiple Comparisons of Means: Tukey Contrasts
##
##
## Fit: lmer(formula = d15N ~ -1 + bodypart + (1 | Site), data = A)
##
## Linear Hypotheses:
## Estimate Std. Error z value Pr(>|z|)
## Shoots - Roots == 0 0.9571 0.1228 7.794 6.44e-15 ***
## ---
## Signif. codes: 0 '***' 0.001 '**' 0.01 '*' 0.05 '.' 0.1 ' ' 1
## (Adjusted p values reported -- single-step method)

### 3: General model: trophic niche prediction across species ----------------------------------------------------------------------------------

### Load data without plants into 'fast-name' object
A <- Data_raw_tabular_plants %>%
 dplyr::select(c(15,16,18,17,13,1:5,7:14,19:24)) %>%
 filter(Group!="Plantae")

### LMM model for baseline-calibrated D13C of soil fauna depending on root/shoot signature and plant species
# site is random effect
model13C_iso1 <- lmer(D13C~1+D13Croots+D13Cshoots+Art+(1|Site)+(1|Taxon),A)
model13C_iso2 <- lmer(D13C~1+D13Croots+D13Cshoots+(1|Site)+(1|Taxon),A)
model13C_iso3 <- lmer(D13C~1+D13Cshoots+Art+(1|Site)+(1|Taxon),A)
model13C_iso4 <- lmer(D13C~1+D13Croots+Art+(1|Site)+(1|Taxon),A)
model13C_iso5 <- lmer(d13C~1+D13Croots+(1|Site)+(1|Taxon),A)
model13C_iso6 <- lmer(d13C~1+D13Cshoots+(1|Site)+(1|Taxon),A)
model13C_iso7 <- lmer(d13C~1+Art+(1|Site)+(1|Taxon),A)
model13C_iso8 <- lmer(d13C~1+(1|Site)+(1|Taxon),A)

model13C_iso_list <- list(model13C_iso1,model13C_iso2,model13C_iso3,model13C_iso4,model13C_iso5,
 model13C_iso6,model13C_iso7,model13C_iso8)

# Compare models with AICc
# The model without factors is selected
AICc <- lapply(model13C_iso_list,AICc)
Model <- lapply(model13C_iso_list,function(x) as.character(c(formula(x))))
model13Cselect_iso <- as.data.frame(cbind(unlist(Model),unlist(AICc)))
model13Cselect_iso <- model13Cselect_iso[order(model13Cselect_iso$V2),]
model13Cselect_iso

## V1
## 8 d13C ~ 1 + (1 | Site) + (1 | Taxon)
## 5 d13C ~ 1 + D13Croots + (1 | Site) + (1 | Taxon)
## 4 D13C ~ 1 + D13Croots + Art + (1 | Site) + (1 | Taxon)
## 6 d13C ~ 1 + D13Cshoots + (1 | Site) + (1 | Taxon)
## 2 D13C ~ 1 + D13Croots + D13Cshoots + (1 | Site) + (1 | Taxon)
## 7 d13C ~ 1 + Art + (1 | Site) + (1 | Taxon)
## 3 D13C ~ 1 + D13Cshoots + Art + (1 | Site) + (1 | Taxon)
## 1 D13C ~ 1 + D13Croots + D13Cshoots + Art + (1 | Site) + (1 | Taxon)
## V2
## 8 699.551112748439
## 5 702.863766484343
## 4 704.311808086389
## 6 704.815886508635
## 2 704.860689195247
## 7 706.237061750204
## 3 706.36049475784
## 1 707.269595126952

### LMM model for baseline-calibrated D13C of soil fauna depending on size group, fallow age and plant species
# site is random effect
model13C_1 <- lmer(D13C~1+Size_group+(1|Site)+(1|Taxon),A)
model13C_2 <- lmer(D13C~1+Trophic_group+(1|Site)+(1|Taxon),A)
model13C_3 <- lmer(D13C~1+Fallow_age+(1|Site)+(1|Taxon),A)
model13C_4 <- lmer(D13C~1+Art+(1|Site)+(1|Taxon),A)
model13C_5 <- lmer(D13C~1+(1|Site)+(1|Taxon),A)

model13C_list1 <- c(model13C_1,model13C_2,model13C_3,model13C_4,model13C_5)

# Compare models
# The model with fallow age is selected
AICc <- lapply(model13C_list1,AICc)
Model <- lapply(model13C_list1,function(x) as.character(c(formula(x))))
model13Cselect <- as.data.frame(cbind(unlist(Model),unlist(AICc)))
model13Cselect <- model13Cselect[order(model13Cselect$V2),]
model13Cselect

## V1 V2
## 3 D13C ~ 1 + Fallow_age + (1 | Site) + (1 | Taxon) 694.824853819153
## 1 D13C ~ 1 + Size_group + (1 | Site) + (1 | Taxon) 696.124034092829
## 5 D13C ~ 1 + (1 | Site) + (1 | Taxon) 696.499589089589
## 2 D13C ~ 1 + Trophic_group + (1 | Site) + (1 | Taxon) 696.571768355004
## 4 D13C ~ 1 + Art + (1 | Site) + (1 | Taxon) 703.165579747939

# Test if the additional factors will improve it (but not trophic group, since we had no predatory mesofauna)
model13C_1 <- lmer(D13C~1+Fallow_age+(1|Site)+(1|Taxon),A)
model13C_2 <- lmer(D13C~1+Fallow_age+Art+(1|Site)+(1|Taxon),A)
model13C_3 <- lmer(D13C~1+Fallow_age+Size_group+(1|Site)+(1|Taxon),A)
model13C_4 <- lmer(D13C~1+Fallow_age+Size_group+Art+(1|Site)+(1|Taxon),A)
model13C_5 <- lmer(D13C~1+(Fallow_age+Art)^2+(1|Site)+(1|Taxon),A)
model13C_6 <- lmer(D13C~1+(Fallow_age+Size_group)^2+(1|Site)+(1|Taxon),A)
model13C_7 <- lmer(D13C~1+(Fallow_age+Size_group+Art)^2+(1|Site)+(1|Taxon),A)

model13C_list2 <- c(model13C_1,model13C_2,model13C_3,model13C_4,model13C_5,model13C_6,model13C_7)

# Compare models
# The model with size group and fallow age with interaction is selected (model13C_6)
AICc <- lapply(model13C_list2,AICc)
Model <- lapply(model13C_list2,function(x) as.character(c(formula(x))))
model13Cselect <- as.data.frame(cbind(unlist(Model),unlist(AICc)))
model13Cselect <- model13Cselect[order(model13Cselect$V2),]
model13Cselect

## V1
## 6 D13C ~ 1 + (Fallow_age + Size_group)^2 + (1 | Site) + (1 | Taxon)
## 3 D13C ~ 1 + Fallow_age + Size_group + (1 | Site) + (1 | Taxon)
## 1 D13C ~ 1 + Fallow_age + (1 | Site) + (1 | Taxon)
## 4 D13C ~ 1 + Fallow_age + Size_group + Art + (1 | Site) + (1 | Taxon)
## 2 D13C ~ 1 + Fallow_age + Art + (1 | Site) + (1 | Taxon)
## 5 D13C ~ 1 + (Fallow_age + Art)^2 + (1 | Site) + (1 | Taxon)
## 7 D13C ~ 1 + (Fallow_age + Size_group + Art)^2 + (1 | Site) + (1 | Taxon)
## V2
## 6 692.85210270622
## 3 694.415443455731
## 1 694.824853819153
## 4 701.074645886007
## 2 701.466919769835
## 5 702.828504945737
## 7 704.443656399137

# Write the model estimates with confidence intervals
selected_model <- model13C_list2[which.min(AICc)][[1]] # the model selected
rownames(anova(selected_model))

## [1] "Fallow_age" "Size_group" "Fallow_age:Size_group"

lsmeans(selected_model,rownames(anova(selected_model))[1], adjust="none")

## NOTE: Results may be misleading due to involvement in interactions

## Fallow_age lsmean SE df lower.CL upper.CL
## Young fallow 1.726 0.509 6.85 0.517 2.93
## Old fallow 0.889 0.495 6.15 -0.315 2.09
##
## Results are averaged over the levels of: Size_group
## Degrees-of-freedom method: kenward-roger
## Confidence level used: 0.95

lsmeans(selected_model,rownames(anova(selected_model))[2], adjust="none")

## NOTE: Results may be misleading due to involvement in interactions

## Size_group lsmean SE df lower.CL upper.CL
## Macrofauna 0.976 0.390 8.37 0.0847 1.87
## Mesofauna 1.638 0.515 15.52 0.5436 2.73
##
## Results are averaged over the levels of: Fallow_age
## Degrees-of-freedom method: kenward-roger
## Confidence level used: 0.95

lsmeans <- as.data.frame(lsmeans(selected_model,rownames(anova(selected_model))[c(1,2)], adjust="none"))
lsmeans[,-c(length(lsmeans)-5:length(lsmeans))] <- round(lsmeans[,-c(length(lsmeans)-5:length(lsmeans))],2)
lsmeans

## Fallow_age Size_group lsmean SE df lower.CL upper.CL
## 1 Young fallow Macrofauna 1.62 0.50 6.31 0.42 2.83
## 2 Old fallow Macrofauna 0.33 0.50 6.41 -0.88 1.54
## 3 Young fallow Mesofauna 1.83 0.66 15.75 0.43 3.23
## 4 Old fallow Mesofauna 1.45 0.62 12.27 0.10 2.79

write.csv(lsmeans,"model13Cselect_summary.csv")

# Test the differences with Tukey contrasts
A$Interaction <- interaction(A$Size_group,A$Fallow_age)
model13C_6_interaction <- lmer(D13C~1+Interaction+(1|Site)+(1|Taxon),A)
posthoc <- glht(model13C_6_interaction, linfct=mcp(Interaction="Tukey"))
summary(posthoc)

##
## Simultaneous Tests for General Linear Hypotheses
##
## Multiple Comparisons of Means: Tukey Contrasts
##
##
## Fit: lmer(formula = D13C ~ 1 + Interaction + (1 | Site) + (1 | Taxon),
## data = A)
##
## Linear Hypotheses:
## Estimate Std. Error
## Mesofauna.Young fallow - Macrofauna.Young fallow == 0 0.2055 0.5695
## Macrofauna.Old fallow - Macrofauna.Young fallow == 0 -1.2934 0.6261
## Mesofauna.Old fallow - Macrofauna.Young fallow == 0 -0.1750 0.7922
## Macrofauna.Old fallow - Mesofauna.Young fallow == 0 -1.4988 0.8235
## Mesofauna.Old fallow - Mesofauna.Young fallow == 0 -0.3805 0.7510
## Mesofauna.Old fallow - Macrofauna.Old fallow == 0 1.1183 0.5314
## z value Pr(>|z|)
## Mesofauna.Young fallow - Macrofauna.Young fallow == 0 0.361 0.980
## Macrofauna.Old fallow - Macrofauna.Young fallow == 0 -2.066 0.145
## Mesofauna.Old fallow - Macrofauna.Young fallow == 0 -0.221 0.995
## Macrofauna.Old fallow - Mesofauna.Young fallow == 0 -1.820 0.236
## Mesofauna.Old fallow - Mesofauna.Young fallow == 0 -0.507 0.948
## Mesofauna.Old fallow - Macrofauna.Old fallow == 0 2.105 0.134
## (Adjusted p values reported -- single-step method)

### LMM model for d15N of soil fauna depending on root/shoot signature and plant species
# site is random effect
model15N_iso1 <- lmer(D15N~1+D15Nroots+(1|Site)+(1|Taxon),A)
model15N_iso2 <- lmer(D15N~1+D15Nshoots+(1|Site)+(1|Taxon),A)
model15N_iso3 <- lmer(D15N~1+Art+(1|Site)+(1|Taxon),A)
model15N_iso4 <- lmer(D15N~1+(1|Site)+(1|Taxon),A)

model15N_iso_list <- c(model15N_iso1,model15N_iso2,model15N_iso3,model15N_iso4)

# Compare models with AICc
# The model with shoots is selected
AICc <- lapply(model15N_iso_list,AICc)
Model <- lapply(model15N_iso_list,function(x) as.character(c(formula(x))))
model15Nselect_iso <- as.data.frame(cbind(unlist(Model),unlist(AICc)))
model15Nselect_iso <- model15Nselect_iso[order(model15Nselect_iso$V2),]
model15Nselect_iso

## V1 V2
## 4 D15N ~ 1 + (1 | Site) + (1 | Taxon) 788.835693138475
## 2 D15N ~ 1 + D15Nshoots + (1 | Site) + (1 | Taxon) 789.088342557904
## 1 D15N ~ 1 + D15Nroots + (1 | Site) + (1 | Taxon) 791.506383445198
## 3 D15N ~ 1 + Art + (1 | Site) + (1 | Taxon) 792.454461898582

### LMM model for D15N of soil fauna depending on size group, fallow age and plant species. We include shoots
# site is random effect
model15N_1 <- lmer(D15N~1+Size_group+(1|Site)+(1|Taxon),A)
model15N_2 <- lmer(D15N~1+Trophic_group+(1|Site)+(1|Taxon),A)
model15N_3 <- lmer(D15N~1+Fallow_age+(1|Site)+(1|Taxon),A)
model15N_4 <- lmer(D15N~1+Art+(1|Site)+(1|Taxon),A)
model15N_5 <- lmer(D15N~1+(1|Site)+(1|Taxon),A)

model15N_list1 <- c(model15N_1,model15N_2,model15N_3,model15N_4,model15N_5)

# Compare models
# The model with trophic group is selected
AICc <- lapply(model15N_list1,AICc)
Model <- lapply(model15N_list1,function(x) as.character(c(formula(x))))
model15Nselect <- as.data.frame(cbind(unlist(Model),unlist(AICc)))
model15Nselect <- model15Nselect[order(model15Nselect$V2),]
model15Nselect

## V1 V2
## 2 D15N ~ 1 + Trophic_group + (1 | Site) + (1 | Taxon) 781.800447768655
## 1 D15N ~ 1 + Size_group + (1 | Site) + (1 | Taxon) 788.392352394194
## 5 D15N ~ 1 + (1 | Site) + (1 | Taxon) 788.835693138475
## 3 D15N ~ 1 + Fallow_age + (1 | Site) + (1 | Taxon) 789.394527506941
## 4 D15N ~ 1 + Art + (1 | Site) + (1 | Taxon) 792.454461898582

# Test if the additional factors will improve it (but not size group, since we had no predatory mesofauna)
model15N_1 <- lmer(D15N~1+Trophic_group+(1|Site)+(1|Taxon),A)
model15N_2 <- lmer(D15N~1+Trophic_group+Art+(1|Site)+(1|Taxon),A)
model15N_3 <- lmer(D15N~1+Trophic_group+Fallow_age+(1|Site)+(1|Taxon),A)
model15N_4 <- lmer(D15N~1+Trophic_group+Art+Fallow_age+(1|Site)+(1|Taxon),A)
model15N_5 <- lmer(D15N~1+(Trophic_group+Art)^2+(1|Site)+(1|Taxon),A)
model15N_6 <- lmer(D15N~1+(Trophic_group+Fallow_age)^2+(1|Site)+(1|Taxon),A)
model15N_7 <- lmer(D15N~1+(Trophic_group+Art+Fallow_age)^2+(1|Site)+(1|Taxon),A)

model15N_list2 <- c(model15N_1,model15N_2,model15N_3,model15N_4,model15N_5,model15N_6,model15N_7)

# Compare models
# The model with trophic group only is still selected (model15N_1)
AICc <- lapply(model15N_list2,AICc)
Model <- lapply(model15N_list2,function(x) as.character(c(formula(x))))
model15Nselect <- as.data.frame(cbind(unlist(Model),unlist(AICc)))
model15Nselect <- model15Nselect[order(model15Nselect$V2),]
model15Nselect

## V1
## 1 D15N ~ 1 + Trophic_group + (1 | Site) + (1 | Taxon)
## 3 D15N ~ 1 + Trophic_group + Fallow_age + (1 | Site) + (1 | Taxon)
## 6 D15N ~ 1 + (Trophic_group + Fallow_age)^2 + (1 | Site) + (1 | Taxon)
## 2 D15N ~ 1 + Trophic_group + Art + (1 | Site) + (1 | Taxon)
## 4 D15N ~ 1 + Trophic_group + Art + Fallow_age + (1 | Site) + (1 | Taxon)
## 5 D15N ~ 1 + (Trophic_group + Art)^2 + (1 | Site) + (1 | Taxon)
## 7 D15N ~ 1 + (Trophic_group + Art + Fallow_age)^2 + (1 | Site) + (1 | Taxon)
## V2
## 1 781.800447768655
## 3 782.435650506142
## 6 783.912960230465
## 2 785.40129920958
## 4 786.173702113378
## 5 787.644268400552
## 7 790.429282932318

# Write the model estimates with confidence intervals
selected_model <- model15N_list2[which.min(AICc)][[1]] # the model selected
rownames(anova(selected_model))

## [1] "Trophic_group"

lsmeans <- as.data.frame(lsmeans(selected_model,rownames(anova(selected_model))[1], adjust="none"))
lsmeans[,-c(length(lsmeans)-5:length(lsmeans))] <- round(lsmeans[,-c(length(lsmeans)-5:length(lsmeans))],2)
lsmeans

## Trophic_group lsmean SE df lower.CL upper.CL
## 1 Decomposers 4.96 0.53 14.53 3.83 6.10
## 2 Predators/omnivores 7.09 0.48 13.29 6.05 8.13

write.csv(lsmeans,"model15Nselect_summary.csv")

# Test the differences with Tukey contrasts
posthoc <- glht(selected_model, linfct=mcp(Trophic_group="Tukey"))
summary(posthoc)

##
## Simultaneous Tests for General Linear Hypotheses
##
## Multiple Comparisons of Means: Tukey Contrasts
##
##
## Fit: lmer(formula = D15N ~ 1 + Trophic_group + (1 | Site) + (1 | Taxon),
## data = A)
##
## Linear Hypotheses:
## Estimate Std. Error z value
## Predators/omnivores - Decomposers == 0 2.126 0.650 3.271
## Pr(>|z|)
## Predators/omnivores - Decomposers == 0 0.00107 **
## ---
## Signif. codes: 0 '***' 0.001 '**' 0.01 '*' 0.05 '.' 0.1 ' ' 1
## (Adjusted p values reported -- single-step method)

### 4: Food-web analysis (species-site) ----------------------------------------------------------------------------------
# Isotopic metrics based on Cucherousset and Villeger 2015 10.1016/j.ecolind.2015.03.032 were calculated
# using the script "si_div.R" (Supplement of Cucherousset and Villeger 2015)
# METRICS:
# minimum, maximum, range and mean values for each isotopic axis
# (e.g. 'min_d13C', 'max_d13C', 'range_d13C', 'IPos_d13C' for d13C)
# and 5 multidimensional indices: isotopic richness ('IRic'), isotopic evenness ('IEve')
# isotopic divergence ('IDiv'), isotopic dispersion ('IDis'), isotopic uniqueness ('IUni').

# Load data
metrics_Salamon <- read.csv(file = "metrics_Salamon.csv", header = T,
 dec = ".", # the decimal point
 sep = ",") # the separator symbol

##### Now we can run tests if metrics depend on the fallow age and species
# transform in the long-table format (and ensure value to be numeric and not factor)
metrics_melted <- melt(metrics_Salamon,id.vars = c("Fallow_age","Art","Site"))
str(metrics_melted)

## 'data.frame': 234 obs. of 5 variables:
## $ Fallow_age: Factor w/ 2 levels "Old fallow","Young fallow": 1 1 2 2 2 1 1 1 2 2 ...
## $ Art : Factor w/ 3 levels "Bromus sterilis",..: 1 1 1 1 1 2 2 2 2 2 ...
## $ Site : Factor w/ 6 levels "O1","O2","O3",..: 2 3 4 5 6 1 2 3 4 5 ...
## $ variable : Factor w/ 13 levels "min_d15N","min_d13C",..: 1 1 1 1 1 1 1 1 1 1 ...
## $ value : num 0.224 0.0928 0.1946 0.156 0.2788 ...

# check the data distribution
ggplot(metrics_melted,aes(x=value))+
 geom_histogram(bins = 8)+
 facet_wrap(~variable,scales='free')


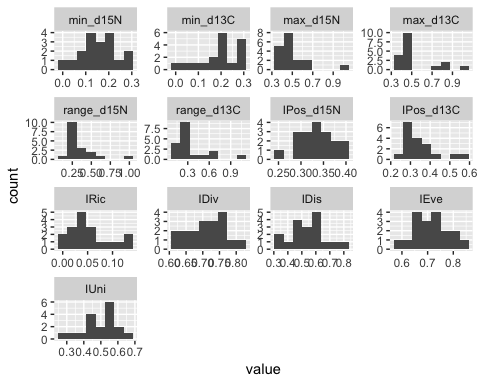


# in most of the cases, the data distributed close to normal so we can go on with lm
# loop through the metrics and run lmer to test the effect of fallow age and species
output_modelmetricselect <- as.data.frame(matrix(ncol=3,nrow=0))
output_modelmetricselectSummary <- as.data.frame(matrix(ncol=1,nrow=0))
names(output_modelmetricselectSummary) <- c("lsmean")
for(i in 1:length(unique(metrics_melted$variable))){
 loop <- subset(metrics_melted, variable==unique(metrics_melted$variable)[i])

 # run candidate models
 modelmetric1 <- lmer(value~1+(1|Site),loop)
 modelmetric2 <- lmer(value~1+Art+(1|Site),loop)
 modelmetric3 <- lmer(value~1+Fallow_age+(1|Site),loop)
 modelmetric4 <- lmer(value~1+Art+Fallow_age+(1|Site),loop)

 # select a model, record the selection
 modelmetriclist <- c(modelmetric1,modelmetric2,modelmetric3,modelmetric4)
 AICc <- lapply(modelmetriclist,AICc)
 Model <- lapply(modelmetriclist,function(x) as.character(c(formula(x))))
 modelmetricselect <- as.data.frame(cbind(as.character(Model),as.numeric(AICc)),stringsAsFactors=F)
 modelmetricselect$V2 <- as.numeric(modelmetricselect$V2)
 modelmetricselect <- modelmetricselect[order(modelmetricselect$V2),]
 modelmetricselect$rank <- seq(1:length(modelmetricselect$V2))
 modelmetricselect <- rbind(c(as.character(loop$variable[1]),NA,NA),modelmetricselect)

 # write to output
 output_modelmetricselect <- rbind(output_modelmetricselect,modelmetricselect)

 # if it's not empty model, write output with confidence intervals
 if(which.min(AICc)!=1){
 selected_model <- modelmetriclist[which.min(AICc)][[1]]
 lsmeans <- as.data.frame(lsmeans(selected_model,rownames(anova(selected_model)), adjust="none"))
 lsmeans[,-c(length(lsmeans)-5:length(lsmeans))] <- round(lsmeans[,-c(length(lsmeans)-5:length(lsmeans))],2)
 lsmeans$Metric <- loop$variable[1]

 # write to output
 output_modelmetricselectSummary <- output_modelmetricselectSummary %>%
 bind_rows(lsmeans)
 }
}

## boundary (singular) fit: see ?isSingular

## boundary (singular) fit: see ?isSingular
## boundary (singular) fit: see ?isSingular
## boundary (singular) fit: see ?isSingular

output_modelmetricselect

## V1 V2 rank
## 1 min_d15N <NA> <NA>
## 11 value ~ 1 + (1 | Site) -30.0929742884801 1
## 3 value ~ 1 + Fallow_age + (1 | Site) -22.9084731273602 2
## 2 value ~ 1 + Art + (1 | Site) -14.2099368837648 3
## 4 value ~ 1 + Art + Fallow_age + (1 | Site) -5.75170944891649 4
## 12 min_d13C <NA> <NA>
## 111 value ~ 1 + (1 | Site) -26.5830968646601 1
## 31 value ~ 1 + Fallow_age + (1 | Site) -20.07654495831 2
## 21 value ~ 1 + Art + (1 | Site) -14.7600923477414 3
## 41 value ~ 1 + Art + Fallow_age + (1 | Site) -6.97981416766497 4
## 13 max_d15N <NA> <NA>
## 112 value ~ 1 + (1 | Site) -5.74144559327737 1
## 32 value ~ 1 + Fallow_age + (1 | Site) 0.528099137585313 2
## 22 value ~ 1 + Art + (1 | Site) 6.04352548475268 3
## 42 value ~ 1 + Art + Fallow_age + (1 | Site) 13.5867964893416 4
## 14 max_d13C <NA> <NA>
## 113 value ~ 1 + (1 | Site) -6.06855913842717 1
## 33 value ~ 1 + Fallow_age + (1 | Site) -2.5340662698968 2
## 23 value ~ 1 + Art + (1 | Site) 6.42620176324289 3
## 43 value ~ 1 + Art + Fallow_age + (1 | Site) 11.2344209056804 4
## 15 range_d15N <NA> <NA>
## 114 value ~ 1 + (1 | Site) 1.55763467752405 1
## 34 value ~ 1 + Fallow_age + (1 | Site) 7.64514563817308 2
## 24 value ~ 1 + Art + (1 | Site) 12.1133644610725 3
## 44 value ~ 1 + Art + Fallow_age + (1 | Site) 19.4746017604235 4
## 16 range_d13C <NA> <NA>
## 115 value ~ 1 + (1 | Site) 3.8252512016156 1
## 35 value ~ 1 + Fallow_age + (1 | Site) 8.14384754756742 2
## 25 value ~ 1 + Art + (1 | Site) 12.7263308232912 3
## 45 value ~ 1 + Art + Fallow_age + (1 | Site) 18.3186534429043 4
## 17 IPos_d15N <NA> <NA>
## 116 value ~ 1 + (1 | Site) -56.307701812709 1
## 36 value ~ 1 + Fallow_age + (1 | Site) -47.9549300151538 2
## 26 value ~ 1 + Art + (1 | Site) -37.7806263873755 3
## 46 value ~ 1 + Art + Fallow_age + (1 | Site) -28.1541283160499 4
## 18 IPos_d13C <NA> <NA>
## 117 value ~ 1 + (1 | Site) -36.8309923715914 1
## 37 value ~ 1 + Fallow_age + (1 | Site) -32.5463273756963 2
## 27 value ~ 1 + Art + (1 | Site) -19.0457843603841 3
## 47 value ~ 1 + Art + Fallow_age + (1 | Site) -13.4873931009669 4
## 19 IRic <NA> <NA>
## 118 value ~ 1 + (1 | Site) -57.1759640323325 1
## 38 value ~ 1 + Fallow_age + (1 | Site) -49.703844804357 2
## 28 value ~ 1 + Art + (1 | Site) -41.3942322494641 3
## 48 value ~ 1 + Art + Fallow_age + (1 | Site) -32.6483867477689 4
## 110 IDiv <NA> <NA>
## 119 value ~ 1 + (1 | Site) -38.3077150914485 1
## 39 value ~ 1 + Fallow_age + (1 | Site) -29.5938104596058 2
## 29 value ~ 1 + Art + (1 | Site) -24.2255039908434 3
## 49 value ~ 1 + Art + Fallow_age + (1 | Site) -14.1457799524357 4
## 120 IDis <NA> <NA>
## 1110 value ~ 1 + (1 | Site) -12.3114552247022 1
## 310 value ~ 1 + Fallow_age + (1 | Site) -5.5593213717513 2
## 210 value ~ 1 + Art + (1 | Site) 0.716403949681681 3
## 410 value ~ 1 + Art + Fallow_age + (1 | Site) 8.74226407610453 4
## 121 IEve <NA> <NA>
## 1111 value ~ 1 + (1 | Site) -33.1155118126257 1
## 311 value ~ 1 + Fallow_age + (1 | Site) -24.8472504680447 2
## 211 value ~ 1 + Art + (1 | Site) -17.9527914217812 3
## 411 value ~ 1 + Art + Fallow_age + (1 | Site) -8.41080380179558 4
## 122 IUni <NA> <NA>
## 1112 value ~ 1 + (1 | Site) -18.7609976551817 1
## 312 value ~ 1 + Fallow_age + (1 | Site) -11.7383284825441 2
## 212 value ~ 1 + Art + (1 | Site) -10.1469916777923 3
## 412 value ~ 1 + Art + Fallow_age + (1 | Site) -1.85059623205543 4

output_modelmetricselectSummary

## [1] lsmean
## <0 rows> (or 0-length row.names)

# Write model selection
write.csv(output_modelmetricselect,"modelmetrics.csv")


### 5: Individual models: trophic niche of species ----------------------------------------------------------------------------------

# Load data into 'fast-name' object
A <- Data_raw_tabular_plants %>%
 dplyr::select(c(15,16,18,17,13,1:5,7:14,19,20)) %>%
 filter(Group!="Plantae")

table(A$Taxon)

##
## Aleocharinae Bromus sterilis Geophilus
## 27 0 12
## Julidae Lasius Lepidocyrtus
## 26 38 3
## Medicago sativa Myrmica Oniscus asellus
## 0 17 13
## Philogalumna crassiclava Philonthus Protaphorura
## 11 12 10
## Punctoribates punctum Solenopsis Taraxacum officinale
## 15 18 0
## Xantholinus
## 7

# Different replicates of different taxa
# Running a loop with best model selection procedure for each taxon
# The loop will run only for well-represented taxa and will write model selection and best model results in tables
output_model13Cspselect <- as.data.frame(matrix(ncol=3,nrow=0))
output_model13CspselectSummary <- as.data.frame(matrix(ncol=1,nrow=0))
names(output_model13CspselectSummary) <- c("lsmean")
output_model13CspselectTest <- as.data.frame(matrix(ncol=3,nrow=0))
for(i in 1:length(unique(A$Taxon))){
loop <- subset(A,Taxon == unique(A$Taxon)[i])
replicates <- as.data.frame(table(loop$Art,loop$Fallow_age)) # check for replicates across treatments
if(length(replicates$Freq[replicates$Freq>1])>4){

 # run candidate models
 model13Csp1 <- lmer(D13C~1+(1|Site),loop)
 model13Csp2 <- lmer(D13C~1+Art+(1|Site),loop)
 model13Csp3 <- lmer(D13C~1+Fallow_age+(1|Site),loop)
 model13Csp4 <- lmer(D13C~1+Art+Fallow_age+(1|Site),loop)

 # select a model, record the selection
 model13Csplist <- c(model13Csp1,model13Csp2,model13Csp3,model13Csp4)
 AICc <- lapply(model13Csplist,AICc)
 Model <- lapply(model13Csplist,function(x) as.character(c(formula(x))))
 model13Cspselect <- as.data.frame(cbind(as.character(Model),as.numeric(AICc)),stringsAsFactors=F)
 model13Cspselect$V2 <- as.numeric(model13Cspselect$V2)
 model13Cspselect <- model13Cspselect[order(model13Cspselect$V2),]
 model13Cspselect$rank <- seq(1:length(model13Cspselect$V2))
 model13Cspselect <- rbind(c(as.character(loop$Taxon[1]),NA,NA),model13Cspselect)

 # write to output
 output_model13Cspselect <- rbind(output_model13Cspselect,model13Cspselect)

 # if it's not empty model, write output with confidence intervals
 if(which.min(AICc)!=1){
 selected_model <- model13Csplist[which.min(AICc)][[1]]
 lsmeans <- as.data.frame(lsmeans(selected_model,rownames(anova(selected_model)), adjust="none"))
 lsmeans[,-c(length(lsmeans)-5:length(lsmeans))] <- round(lsmeans[,-c(length(lsmeans)-5:length(lsmeans))],2)
 lsmeans$Metric <- loop$Taxon[1]

 # write to output
 output_model13CspselectSummary <- output_model13CspselectSummary %>%
 bind_rows(lsmeans)

 # Test the differences with Tukey contrasts
 if(which.min(AICc)==3){
 posthoc <- summary(glht(selected_model, linfct=mcp(Fallow_age="Tukey")))
 posthoc_out <- cbind(as.character(loop$Taxon[1]),"Fallow_age",names(posthoc$test$pfunction()),posthoc$test$pvalues[1])
 output_model13CspselectTest <- rbind(output_model13CspselectTest,posthoc_out,stringsAsFactors=F)
 }
 }
}
}

## boundary (singular) fit: see ?isSingular
## boundary (singular) fit: see ?isSingular
## boundary (singular) fit: see ?isSingular

## Warning in as_lmerModLT(model, devfun): Model may not have converged with 1
## eigenvalue close to zero: 1.5e-10

## Warning in checkConv(attr(opt, "derivs"), opt$par, ctrl =
## control$checkConv, : Hessian is numerically singular: parameters are not
## uniquely determined

## Warning in as_lmerModLT(model, devfun): Model may not have converged with 1
## eigenvalue close to zero: 5.0e-11

## boundary (singular) fit: see ?isSingular
## boundary (singular) fit: see ?isSingular

output_model13Cspselect

## V1 V2 rank
## 1 Julidae <NA> <NA>
## 11 D13C ~ 1 + (1 | Site) 85.4548018613206 1
## 3 D13C ~ 1 + Fallow_age + (1 | Site) 86.5175848769103 2
## 2 D13C ~ 1 + Art + (1 | Site) 89.315346284058 3
## 4 D13C ~ 1 + Art + Fallow_age + (1 | Site) 91.4136802884318 4
## 12 Lasius <NA> <NA>
## 31 D13C ~ 1 + Fallow_age + (1 | Site) 130.080206193391 1
## 41 D13C ~ 1 + Art + Fallow_age + (1 | Site) 133.872047101845 2
## 111 D13C ~ 1 + (1 | Site) 134.043741024955 3
## 21 D13C ~ 1 + Art + (1 | Site) 137.154662783782 4
## 13 Punctoribates punctum <NA> <NA>
## 112 D13C ~ 1 + (1 | Site) 8.79582988261874 1
## 32 D13C ~ 1 + Fallow_age + (1 | Site) 11.0673668689849 2
## 22 D13C ~ 1 + Art + (1 | Site) 13.7270569529469 3
## 42 D13C ~ 1 + Art + Fallow_age + (1 | Site) 17.9962965229092 4
## 14 Aleocharinae <NA> <NA>
## 33 D13C ~ 1 + Fallow_age + (1 | Site) 91.8606512393097 1
## 113 D13C ~ 1 + (1 | Site) 91.8677256920863 2
## 23 D13C ~ 1 + Art + (1 | Site) 95.313351859347 3
## 43 D13C ~ 1 + Art + Fallow_age + (1 | Site) 95.5320085472592 4
## 15 Philonthus <NA> <NA>
## 24 D13C ~ 1 + (1 | Site) 42.6239650394436 1
## 34 D13C ~ 1 + Art + (1 | Site) 45.0605459716382 2
## 44 D13C ~ 1 + Fallow_age + (1 | Site) 46.5209337484148 3
## 5 D13C ~ 1 + Art + Fallow_age + (1 | Site) 52.4464898699394 4

output_model13CspselectSummary

## lsmean Fallow_age SE df lower.CL upper.CL Metric
## 1 3.34 Young fallow 1.01 4.64 0.68 6.00 Lasius
## 2 0.25 Old fallow 0.90 3.29 -2.47 2.96 Lasius
## 3 0.69 Young fallow 0.43 4.08 -0.50 1.89 Aleocharinae
## 4 -0.14 Old fallow 0.39 2.72 -1.47 1.20 Aleocharinae

output_model13CspselectTest

## V1 V2 V3 V4
## 1 Lasius Fallow_age Old fallow - Young fallow 0.0209194551500991
## 2 Aleocharinae Fallow_age Old fallow - Young fallow 0.137848570118083

# Write model selection
write.csv(output_model13Cspselect,"output_model13Cspselect.csv")


### Running a similar loop with best model selection procedure for each taxon for D15N

output_model15Nspselect <- as.data.frame(matrix(ncol=3,nrow=0))
output_model15NspselectSummary <- as.data.frame(matrix(ncol=1,nrow=0))
names(output_model15NspselectSummary) <- c("lsmean")
output_model15NspselectTest <- as.data.frame(matrix(ncol=3,nrow=0))
for(i in 1:length(unique(A$Taxon))){
 loop <- subset(A,Taxon == unique(A$Taxon)[i])
 replicates <- as.data.frame(table(loop$Art,loop$Fallow_age)) # check for replicates across treatments
 if(length(replicates$Freq[replicates$Freq>1])>4){

 # run candidate models
 model15Nsp1 <- lmer(D15N~1+(1|Site),loop)
 model15Nsp2 <- lmer(D15N~1+Art+(1|Site),loop)
 model15Nsp3 <- lmer(D15N~1+Fallow_age+(1|Site),loop)
 model15Nsp4 <- lmer(D15N~1+Art+Fallow_age+(1|Site),loop)

 # select a model, record the selection
 model15Nsplist <- c(model15Nsp1,model15Nsp2,model15Nsp3,model15Nsp4)
 AICc <- lapply(model15Nsplist,AICc)
 Model <- lapply(model15Nsplist,function(x) as.character(c(formula(x))))
 model15Nspselect <- as.data.frame(cbind(as.character(Model),as.numeric(AICc)),stringsAsFactors=F)
 model15Nspselect$V2 <- as.numeric(model15Nspselect$V2)
 model15Nspselect <- model15Nspselect[order(model15Nspselect$V2),]
 model15Nspselect$rank <- seq(1:length(model15Nspselect$V2))
 model15Nspselect <- rbind(c(as.character(loop$Taxon[1]),NA,NA),model15Nspselect)

 # write to output
 output_model15Nspselect <- rbind(output_model15Nspselect,model15Nspselect)

 # if it's not empty model, write output with confidence intervals
 if(which.min(AICc)!=1){
 selected_model <- model15Nsplist[which.min(AICc)][[1]]
 lsmeans <- as.data.frame(lsmeans(selected_model,rownames(anova(selected_model)), adjust="none"))
 lsmeans[,-c(length(lsmeans)-5:length(lsmeans))] <- round(lsmeans[,-c(length(lsmeans)-5:length(lsmeans))],2)
 lsmeans$Metric <- loop$Taxon[1]

 # write to output
 output_model15NspselectSummary <- output_model15NspselectSummary %>%
 bind_rows(lsmeans)

 # Test the differences with Tukey contrasts
 if(which.min(AICc)==3){
 posthoc <- summary(glht(selected_model, linfct=mcp(Fallow_age="Tukey")))
 posthoc_out <- cbind(as.character(loop$Taxon[1]),"Fallow_age",names(posthoc$test$pfunction()),posthoc$test$pvalues[1])
 output_model15NspselectTest <- rbind(output_model15NspselectTest,posthoc_out,stringsAsFactors=F)
 }
 if(which.min(AICc)==2){
 posthoc1 <- summary(glht(selected_model, linfct=mcp(Art="Tukey")))
 posthoc_out <- cbind(as.character(loop$Taxon[1]),"Art",names(posthoc1$test$pfunction()),posthoc1$test$pvalues)
 output_model15NspselectTest <- rbind(output_model15NspselectTest,posthoc_out,stringsAsFactors=F)
 }
 }
 }
}

## boundary (singular) fit: see ?isSingular
## boundary (singular) fit: see ?isSingular
## boundary (singular) fit: see ?isSingular
## boundary (singular) fit: see ?isSingular

## Warning in as_lmerModLT(model, devfun): Model may not have converged with 1
## eigenvalue close to zero: -5.0e-11

## Warning in as_lmerModLT(model, devfun): Model may not have converged with 1
## eigenvalue close to zero: -5.1e-11

## boundary (singular) fit: see ?isSingular
## boundary (singular) fit: see ?isSingular

output_model15Nspselect

## V1 V2 rank
## 1 Julidae <NA> <NA>
## 11 D15N ~ 1 + (1 | Site) 97.8661422062136 1
## 3 D15N ~ 1 + Fallow_age + (1 | Site) 98.0570361515674 2
## 2 D15N ~ 1 + Art + (1 | Site) 101.004132525636 3
## 4 D15N ~ 1 + Art + Fallow_age + (1 | Site) 101.847313539825 4
## 12 Lasius <NA> <NA>
## 111 D15N ~ 1 + (1 | Site) 88.1494715485676 1
## 31 D15N ~ 1 + Fallow_age + (1 | Site) 91.1311686887696 2
## 21 D15N ~ 1 + Art + (1 | Site) 93.5172179386527 3
## 41 D15N ~ 1 + Art + Fallow_age + (1 | Site) 96.851074393561 4
## 13 Punctoribates punctum <NA> <NA>
## 112 D15N ~ 1 + (1 | Site) 30.3193371057828 1
## 32 D15N ~ 1 + Fallow_age + (1 | Site) 30.3237822802435 2
## 22 D15N ~ 1 + Art + (1 | Site) 38.6675721882551 3
## 42 D15N ~ 1 + Art + Fallow_age + (1 | Site) 40.7217940961477 4
## 14 Aleocharinae <NA> <NA>
## 23 D15N ~ 1 + Art + (1 | Site) 109.422351498758 1
## 33 D15N ~ 1 + Fallow_age + (1 | Site) 110.692670569701 2
## 43 D15N ~ 1 + Art + Fallow_age + (1 | Site) 111.012553316939 3
## 113 D15N ~ 1 + (1 | Site) 111.115681367371 4
## 15 Philonthus <NA> <NA>
## 114 D15N ~ 1 + (1 | Site) 67.6687848341169 1
## 34 D15N ~ 1 + Fallow_age + (1 | Site) 68.379620895658 2
## 24 D15N ~ 1 + Art + (1 | Site) 70.5068914527782 3
## 44 D15N ~ 1 + Art + Fallow_age + (1 | Site) 74.961845286927 4

output_model15NspselectSummary

## lsmean Art SE df lower.CL upper.CL Metric
## 1 7.92 Bromus sterilis 0.53 14.40 6.78 9.06 Aleocharinae
## 2 6.32 Medicago sativa 0.56 16.66 5.15 7.50 Aleocharinae
## 3 6.54 Taraxacum officinale 0.60 17.57 5.28 7.79 Aleocharinae

output_model15NspselectTest

## V1 V2 V3
## 1 Aleocharinae Art Medicago sativa - Bromus sterilis
## 2 Aleocharinae Art Taraxacum officinale - Bromus sterilis
## 3 Aleocharinae Art Taraxacum officinale - Medicago sativa
## V4
## 1 0.0712019265796084
## 2 0.154385667435923
## 3 0.958650876439956

# Write model selection
write.csv(output_model15Nspselect,"output_model15Nspselect.csv")


### 6: Individual metrics: niche ranges ----------------------------------------------------------------------------------

# Load data into 'fast-name' object and calculate SDs
A <- Data_raw_tabular_plants %>%
 dplyr::select(c(15,16,18,17,13,1:5,7:14,19,20)) %>%
 filter(Group!="Plantae") %>%
 group_by(Fallow_age,Site,Taxon) %>%
 dplyr::summarise(n = length(D13C),SdD13C = sd(D13C,na.rm=T),SdD15N = sd(D15N,na.rm=T)) %>%
 filter(n > 2)

# SD in D15N are driven by outliers in Staphylinidae
ggplot(A, aes(x=n,y=SdD13C))+geom_point()+stat_smooth(method='lm')


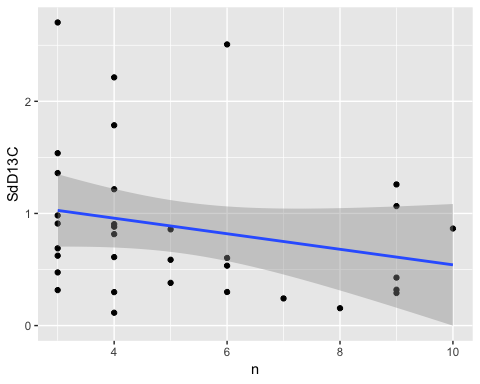


ggplot(A, aes(x=n,y=SdD15N))+geom_point()+stat_smooth(method='lm')


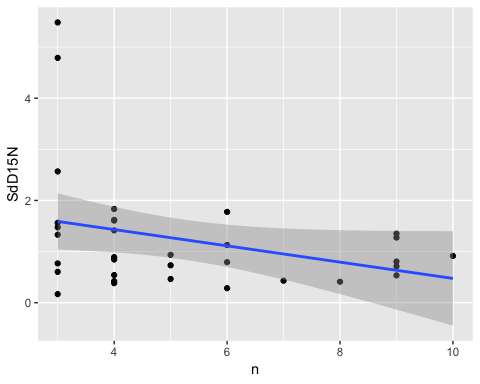


# We remove Staphylinidae with high sd of D15N
A <- A %>%
 filter(SdD15N < 2)
A

## # A tibble: 30 x 6
## # Groups: Fallow_age, Site [6]
## Fallow_age Site Taxon n SdD13C SdD15N
## <fct> <fct> <fct> <int> <dbl> <dbl>
## 1 Young fallow Y1 Aleocharinae 6 0.534 1.13
## 2 Young fallow Y1 Geophilus 3 0.982 1.47
## 3 Young fallow Y1 Julidae 4 1.79 1.83
## 4 Young fallow Y1 Oniscus asellus 5 0.858 0.936
## 5 Young fallow Y1 Protaphorura 3 0.689 0.604
## 6 Young fallow Y1 Punctoribates punctum 7 0.242 0.430
## 7 Young fallow Y2 Aleocharinae 3 2.70 1.33
## 8 Young fallow Y2 Geophilus 4 2.21 0.541
## 9 Young fallow Y2 Julidae 4 1.22 1.62
## 10 Young fallow Y2 Lasius 6 2.51 0.284
## # … with 20 more rows

# Too few points to run individual models, so we run a common model with taxon as random effect
### LMM model for SdD13C depending on fallow age
model13C_sd1 <- lmer(SdD13C~1+(1|Site)+(1|Taxon),A)
model13C_sd2 <- lmer(SdD13C~1+Fallow_age+(1|Site)+(1|Taxon),A)

model13C_sd_list <- c(model13C_sd1,model13C_sd2)

# Compare models with AICc
# The model without fallow age is selected
AICc <- lapply(model13C_sd_list,AICc)
Model <- lapply(model13C_sd_list,function(x) as.character(c(formula(x))))
model13Cselect_sd <- cbind(Model,AICc)
model13Cselect_sd

## Model AICc
## [1,] "SdD13C ~ 1 + (1 | Site) + (1 | Taxon)" 62.99514
## [2,] "SdD13C ~ 1 + Fallow_age + (1 | Site) + (1 | Taxon)" 64.62449

### LMM model for SdD15N depending on fallow age
model15N_sd1 <- lmer(SdD15N~1+(1|Site)+(1|Taxon),A)

## boundary (singular) fit: see ?isSingular

model15N_sd2 <- lmer(SdD15N~1+Fallow_age+(1|Site)+(1|Taxon),A)

## boundary (singular) fit: see ?isSingular

model15N_sd_list <- c(model15N_sd1,model15N_sd2)

# Compare models with AICc
# The model without fallow age is selected
AICc <- lapply(model15N_sd_list,AICc)
Model <- lapply(model15N_sd_list,function(x) as.character(c(formula(x))))
model15Nselect_sd <- cbind(Model,AICc)
model15Nselect_sd

## Model AICc
## [1,] "SdD15N ~ 1 + (1 | Site) + (1 | Taxon)" 49.43487
## [2,] "SdD15N ~ 1 + Fallow_age + (1 | Site) + (1 | Taxon)" 54.21175

### 7: Descriptive table ----------------------------------------------------------------------------------

# Load data into 'fast-name' object and calculate mean values
A <- Data_raw_tabular_plants %>%
 dplyr::select(c(15,16,18,17,13,1:5,7:14,19,20)) %>%
 filter(Group!="Plantae") %>%
 group_by(Fallow_age,Size_group,Trophic_group,Grouptaxon) %>%
 dplyr::summarise(nD13C = length(D13C),meanD13C = mean(D13C,na.rm=T),sdD13C = sd(D13C,na.rm=T),
 nD15N = length(D15N),meanD15N = mean(D15N,na.rm=T),sdD15N = sd(D15N,na.rm=T))

# Represent mean, sd and n as one variable
A$D13C <- paste(round(A$meanD13C,1)," SD ",round(A$sdD13C,1)," (",A$nD13C,")", sep="")
A$D15N <- paste(round(A$meanD15N,1)," SD ",round(A$sdD15N,1)," (",A$nD15N,")", sep="")

# Represent in a table form
A1 <- A %>%
 dplyr::select(Fallow_age,Size_group,Trophic_group,Grouptaxon,D13C,D15N)
A1 <- merge(subset(A1, Fallow_age=='Young fallow'),subset(A1, Fallow_age=='Old fallow'),by=c("Size_group","Trophic_group","Grouptaxon"))

# Write a table
write.csv(A1,"table_descriptives.csv")
